# BATF3 Protects Against Metabolic Syndrome and Maintains Intestinal Epithelial Homeostasis

**DOI:** 10.3389/fimmu.2022.841065

**Published:** 2022-06-22

**Authors:** Hussein Hamade, Jasmine T. Stamps, Dalton T. Stamps, Shyam K. More, Lisa S. Thomas, Anna Y. Blackwood, Nawele L. Lahcene, Sofi L. Castanon, Brenda C. Salumbides, Yosuke Shimodaira, Helen S. Goodridge, Stephan R. Targan, Kathrin S. Michelsen

**Affiliations:** ^1^ F. Widjaja Foundation Inflammatory Bowel & Immunobiology Research Institute, Department of Medicine, Cedars-Sinai Medical Center, Los Angeles, CA, United States; ^2^ Research Division of Immunology, Department of Biomedical Sciences, Cedars-Sinai Medical Center, Los Angeles, CA, United States; ^3^ Board of Governors Regenerative Medicine Institute, Cedars-Sinai Medical Center, Los Angeles, CA, United States

**Keywords:** Metabolic syndrome, intestinal dendritic cells, intestinal permeability, mucosal immunity, BATF3, colitis, hyperglycemia, microbiota

## Abstract

The intestinal immune system and microbiota are emerging as important contributors to the development of metabolic syndrome, but the role of intestinal dendritic cells (DCs) in this context is incompletely understood. BATF3 is a transcription factor essential in the development of mucosal conventional DCs type 1 (cDC1). We show that *Batf3^-/-^* mice developed metabolic syndrome and have altered localization of tight junction proteins in intestinal epithelial cells leading to increased intestinal permeability. Treatment with the glycolysis inhibitor 2-deoxy-D-glucose reduced intestinal inflammation and restored barrier function in obese *Batf3^-/-^* mice. High-fat diet further enhanced the metabolic phenotype and susceptibility to dextran sulfate sodium colitis in *Batf3^-/-^* mice. Antibiotic treatment of *Batf3^-/-^* mice prevented metabolic syndrome and impaired intestinal barrier function. *Batf3^-/-^* mice have altered IgA-coating of fecal bacteria and displayed microbial dysbiosis marked by decreased obesity protective *Akkermansia muciniphila*, and *Bifidobacterium*. Thus, BATF3 protects against metabolic syndrome and preserves intestinal epithelial barrier by maintaining beneficial microbiota.

## Introduction

The prevalence of obesity has been increasing over the past few decades and has been recognized as a major public health challenge. Worldwide, an estimated 2.1 billion adults are overweight of which 600 million are obese (1, 2). Obesity is often associated with the development of metabolic syndrome and increased risk of a variety of chronic diseases including type 2 diabetes, cardiovascular diseases, cancer, and non-alcoholic fatty liver disease (3–8). Metabolic syndrome is a cluster of conditions that includes central obesity, insulin resistance, dyslipidemia, and hypertension (3, 9). While an imbalance of energy intake and expenditure (i.e., diet and lack of exercise) is the major driver of obesity, additional factors contribute to the development of obesity: genetic predisposition, gut microbiota, and host immunity (10–15). The precise mechanisms leading to the development of metabolic syndrome and particularly the involvement of the mucosal immune system are incompletely understood. Growing evidence implicates the intestinal microbiota as an important driver in the development of metabolic syndrome (12, 16, 17). One of the early events in this process has been attributed to a high-fat diet-induced impaired mucosal barrier function, also referred to as “leaky gut” (18–21). As a consequence, intestinal luminal bacteria and their products enter the systemic circulation leading to chronic low-grade inflammation that has been shown to have a profound effect on normal insulin sensitivity (12, 17, 20). Recently, obesity-associated hyperglycemia has also been demonstrated to disturb intestinal epithelial tight-junctions resulting in impaired barrier function (22). That the intestinal microbiota itself are a driver of the development of metabolic syndrome has been supported by the observation that mice raised under germ-free conditions are resistant to the development of obesity and insulin resistance (15, 23, 24). In contrast, transfer of intestinal bacteria from obese to lean mice or reconstitution of germ-free mice induced metabolic syndrome in recipient mice (25). Interestingly, IgA is the most abundant antibody secreted into the intestinal lumen and has been proven to be a key regulator of commensal microbial communities (26). High IgA coating preferentially identifies colitogenic members of the microbiota, that confer susceptibility to the development of colitis, and result in the development of obesity (27, 28). Early recognition of intestinal bacteria crossing from the lumen to the lamina propria is crucial in preventing systemic inflammation and its metabolic consequences and is accomplished by cells of the innate immune system. Toll-like receptors (TLRs) that recognize different bacterial components or downstream signaling molecules, including the inflammasome, have been associated with an increased risk in the development of metabolic syndrome in humans and mice demonstrating the importance of these pathways in the development of metabolic syndrome (14, 15, 29–31). While these studies clearly demonstrate a relationship between diet, intestinal microbiota, immune responses, chronic inflammation, and metabolic syndrome, the exact mechanisms linking the host immune system and intestinal microbiome leading to the development of metabolic syndrome are not well understood.

Intestinal lamina propria conventional dendritic cells (cDCs) play an essential role in mucosal homeostasis, sensing of invading microorganisms, initiation of adaptive immune response, and development of oral tolerance to dietary antigens (32–35). Intestinal cDCs are highly heterogenous and can be subdivided based on the expression of CD103 and CD11b into three major subsets CD103^+^CD11b^-^ (cDC1), CD103^+^CD11b^+^ (cDC2), CD103^-^CD11b^+^, and one minor CD103^-^ CD11b^-^ cDC subset (33, 36–40). While the development of all these subsets is dependent on the transcription factors Zbtb46 and FLT3L, each subset is also dependent on specific transcription factors. Developmentally, cDC type 1 (cDC1) are dependent on the expression of the transcription factors BATF3, IRF8, and ID2 while cDC type 2 (cDC2) are dependent on IRF4, KLF4, and Notch-2 (38, 41, 42). Accordingly, *Batf3^-/-^* mice lack CD8α^+^ DCs in lymphoid organs and CD103^+^ cDC1s in non-lymphoid organs, particularly in the intestine (43–45). Intestinal CD103^+^ cDC1 are important for the development of oral tolerance to dietary antigens and the induction of FoxP3^+^ regulatory T cells (Tregs). However, *Batf3^-/-^* mice do not develop spontaneous intestinal inflammation and maintain a normal population of Tregs in the lamina propria and mesenteric lymph nodes (MLN), suggesting a redundant role of DC subsets in maintaining intestinal homeostasis (43). The mechanisms by which intestinal DC subsets influence intestinal barrier function and homeostasis in the context of the development of metabolic syndrome remain unclear.

In this study, we investigated the role of BATF3 in maintaining intestinal barrier and development of metabolic syndrome. Here, we demonstrate that BATF3-deficiency leads to the development of metabolic syndrome as characterized by insulin resistance, blood glucose and serum insulin levels, increased body weight and white adipocyte size, and development of hepatosteatosis. Hyperinsulinemia and hypercholesteremia were the first metabolic changes observed in lean *Batf3^-/-^* mice and were associated with a decrease of IgA^high^ coated fecal bacteria and intestinal dysbiosis. 16S ribosomal RNA (rRNA) sequencing revealed a significant decrease in the abundance of *Akkermansia muciniphila*, *Mucispirillum schaedleri*, and *Bifidobacterium* and an increased abundance of *Bacteroides* sp. in non-obese *Batf3^-/-^* mice. Mechanistically, we observed altered cellular localization of the tight-junction proteins occludin-1, ZO-1, and claudin-2 in intestinal epithelial cells leading to increased intestinal permeability in obese *Batf3^-/-^* mice. Moreover, intestinal enteroids generated from *Batf3^-/-^* mice had reduced regenerative potential compared to WT mice. Impaired intestinal barrier function was associated with an increase in the percentage of lamina propria cDC2 and CD11c^+^ mononuclear phagocytes (MNPs) and increased expression of IL-1β, IL-18, and TNFα. Treatment of obese *Batf3^-/-^* mice with the glycolysis inhibitor 2-deoxy-D-glucose (2-DG) improved intestinal barrier function and reversed the intestinal inflammatory phenotype to WT levels. In addition, treatment with broad-spectrum antibiotics prevented the development of metabolic syndrome, low-grade intestinal inflammation, impaired intestinal barrier function, and normalized insulin tolerance in *Batf3^-/-^* mice suggesting that intestinal microbiota are essential in the development of metabolic syndrome and the pro-inflammatory phenotype in *Batf3^-/-^* mice. Our data suggest that deficiency of the transcription factor BATF3 results in phenotypical changes of IgA-coating of bacteria, microbial dysbiosis, and impairs intestinal epithelial barrier function, leading to low-grade inflammation that contributes to the development of metabolic syndrome.

## Materials and Methods

### Mice

C57BL/6J, *Batf3^-/-^* (B6.129S(C)-*Batf3^tm1kmm^*/J; stock # 013755), and *Irf8^-/-^* (B6(Cg)-*Irf8^tm1.2Hm^*/J; stock # 018298) mice were purchased from the Jackson Laboratory. *Batf3^-/-^* were backcrossed to C57BL/6J mice to establish WT and *Batf3^-/-^* colonies from the heterozygote littermates. Mice were maintained under SPF conditions. All animal studies were approved by the Cedars-Sinai Medical Center Animal Care and Use Committee (IACUC protocol # 5487).

### Insulin Tolerance Test (ITT)

ITT was performed on 8- or 16-week-old mice. After overnight fasting, blood glucose levels were measured *via* tail puncture using a glucose monitoring system (Easy touch). Mice were injected intraperitoneal (i.p.) with human insulin (1 U/kg) (Millipore Sigma). Blood glucose concentrations were recorded at 0, 15, 30, 60, and 120 min. after i.p. injection. Homeostatic Model Assessment for Insulin Resistance (HOMA-IR) was calculated using fasting blood glucose and serum insulin values in the following equation: HOMA-IR = [(fasting insulin) x (fasting glucose)/405] (46).

### Depletion of the Gut Microbiota by Antibiotic Treatment

Mice received antibiotic treatment from the time of weaning until 16 weeks of age. A combination of four antibiotics (1 g/l ampicillin, 500 mg/l vancomycin, 1 g/l neomycin sulfate, 1 g/l metronidazole) was added to the drinking water and changed once per week (47, 48).

### 2-Deoxy-D-Glucose (2-DG) Treatment

Sixteen-week-old mice were treated twice daily with i.p. injection of 2-DG (5 mg/mouse; Sigma-Aldrich) for 10 days as previously described (49).

### Quantification of Food Intake

Food consumption was measured over a 24 h time period twice a week and normalized to the number of mice in the cages per day. A minimum of five cages by genotype were measured every week from 8 to 16 weeks of age.

### Serum Insulin and Total Cholesterol Measurements

Eight- or 16-week-old mice were fasted overnight, and blood was collected by retro-orbital bleeding. Serum was stored at -80°C until use. Serum insulin concentrations were measured by ELISA according to the manufacturer’s instructions (Crystal Chem, Elk Grove Village, IL, USA). Serum total cholesterol concentrations were measured using cholesterol fluorometric assay according to the manufacturer’s instructions (Cayman Chemical, Ann Arbor, MI, USA).

### FITC-Dextran Assay

*In vivo* colonic epithelial barrier permeability was measured using FITC-Dextran (average MW 3000-5000; Sigma-Aldrich). Briefly, mice were fasted overnight and gavaged with FITC-Dextran (50 mg/100 g), and serum was collected 3 hours after gavage. Fluorescence was measured at an excitation wavelength of 485 nm and emission wavelength of 538 nm on a SpectraMax i3 spectrometer (Molecular Devices).

### Isolation of Mononuclear Cells From Lamina Propria and Flow Cytometry Analysis

Lamina propria mononuclear cells (LPMC) were isolated from the large intestine as previously described (50). Single cell suspensions were stained with antibodies or corresponding isotype controls. All antibodies were purchased from Thermo Fisher Scientific: anti-CD11c (N418), anti-MHC-II (M5/114.15.2), anti-CD11b (M1/70), anti-CD103 (2E7), and anti-F4/80 (BM8). Cells were acquired by flow cytometry using an LSR II analyzer (BD Biosciences). Samples were analyzed using FlowJo software (TreeStar Inc.). The following gating strategy was used to analyze LPMC: immune cells were gated based on forward and side scatter excluding cell aggregates. Then DC subsets were gated on MHCII^+^CD11c^+^ and cDC1 and cDC2 identified as CD103^+^CD11b^-^ and CD103^+^ CD11b^+^, respectively. CD11c^+^ MNPs were gated on CD11b^+^CD11c^+^ and identified as F4/80^+^.

### Isolation of Intestinal Epithelial Cells (IEC)

Small intestinal IEC were isolated from WT or *Batf3^-/-^* mice as described (50). Briefly, after cleaning, small intestines were cut in small pieces and incubated in HBSS/5 mM EDTA/1 mM DTT/5% FCS in a shaking water bath for 20 min at 37°C. Tissue pieces were then vortexed and filtered through cell strainers. IEC were stained with EpCam (Invitrogen; clone: G8.8) and CD45 (eBioscience; clone: 104) antibodies and sorted as CD45^-^EpCam^+^ on a FACSAria III cell sorter. RNA was isolated and mRNA expression of *Batf3, Irf8*, and *Muc2* was analyzed by qPCR as described below.

### Analysis of Adipocyte Size

Adipocyte sizes were quantified as described (51). One hundred adipocytes were analyzed per mouse using ImageJ software.

### Acute Dextran Sulfate Sodium (DSS) Colitis

Colitis was induced in female mice with 2.6% DSS drinking water *ad libitum* for 7 days followed by one day of regular drinking water before being euthanized. Body weights were recorded daily during colitis induction and recovery phases of the experiment. H & E sections of cecum, colon, and rectum were scored by a trained observer blinded to the genotypes and treatments as described (52).

### High Fat Diet (HFD)

Female and male WT and *Batf3^-/-^* mice were either maintained on normal chow diet (25% kcal from fat; LabDiet, 5LJ5) or HFD (60% kcal from fat; Envigo, TD.06414) starting at 8 weeks of age to 16 weeks of age.

### Immunofluorescence Staining and Confocal Microscopy

Intestines were excised from 8-week-old mice and immediately fixed in 10% neutral buffered formalin followed by paraffin-embedding. Tissue sections were deparaffinized, boiled in citrate buffer (10 mM Sodium Citrate, 0.05% Tween-20; pH 6.0) for antigen-retrieval, blocked with hydrogen peroxide, and incubated with primary antibodies against Occludin (Thermo Fisher Scientific, 40-4700), ZO-1 (Thermo Fisher Scientific, 61-7300), or Claudin-2 (Abcam, ab53032) overnight. Fluorophore-conjugated secondary antibodies were incubated for 1 h. Images were captured with TCS SP5 X confocal microscope (Leica). Whole mount enteroid staining was performed as previously described (53). Briefly, enteroids were fixed in 4% paraformaldehyde, blocked with PBS/0.1% Triton-x-100/0.2% BSA (enteroid wash buffer), and incubated either with rabbit anti-ZO-1 (1:100), rabbit anti-Claudin-2 (1:200), or rabbit anti-occludin (1:50) in combination with goat anti-E-cadherin (R&D Systems, AF648, 1:200) antibodies overnight at 4°C. The enteroids were incubated overnight with donkey anti-goat AF 594 (Life technology, A11058, 1:500) and Donkey anti-rabbit Dylight 650 (Abcam, ab96922, 1:500) followed by nuclei staining with Hoechst 33342 dye (Thermo Fisher Scientific, 62249). The enteroids were mounted on glass slides using fructose-glycerol solution. Images were acquired using Leica Stellaris 8 confocal microscope and fluorescence intensity was analyzed using Fiji ImageJ software.

### ELISA

For colonic secreted cytokines, excised colons were flushed with ice-cold PBS and 5-10 mm tissue sections were cultured in RPMI-1640 medium supplemented with 10% FBS, 50 μg/ml gentamicin, 0.25 μg/ml amphotericin B, 100 U/ml Penicillin G, 0.1 mg/ml Streptomycin, and 50 μM β-mercaptoethanol. Supernatants from 24 h cultures were removed, cleared of debris by centrifugation, and stored at -80°C until analysis. Cytokine concentration in culture supernatants were assayed by ELISA for murine IL-1β (eBioscience), and IL-18 (RayBiotech) according to the manufacturer’s instructions. Concentrations of secreted cytokines were normalized to the dry weight of the tissue sections.

### Quantitative RT-PCR (qPCR)

Total RNA was isolated using RNeasy kits and reverse transcribed into cDNA with Omniscript RT kit (both Qiagen). QPCR was performed using the Mastercycler^®^ ep realplex^2^ System (Eppendorf). Platimum^®^ Quantitative PCR SuperMix-UDG (Invitrogen) and TaqMan probes and primers were used for *Actb, Il1b, Tnfa, Il6*, and SsoAdvanced Universal SYBR^®^ Green Supermix (Bio-Rad) was used for *Batf3*, *Muc2*, *Irf8*, 16S rRNA, *A. muciniphila, M. schaedleri, Bifidobacterium* spp., and *Bacteroides* sp. (**Supplemental Table**). mRNA expression of target genes was normalized to the expression of *Actb*. The relative gene expression was calculated by the 2^-^ΔΔ^Ct^ method.

### Extraction of Fecal DNA, 16S rRNA Sequencing, and Analysis of Fecal Microbiota by Quantitative PCR (qPCR)

Fecal samples were collected from female and male 8-week-old WT and *Batf3^-/-^* mice, snap-frozen, and stored at -80°C. DNA was isolated using the DNeasy PowerSoil DNA Isolation Kit (Qiagen) following the manufacturer’s protocol. The V4 region of the 16S gene was amplified and barcoded using 515f/806r primers and 250x2 bp sequencing was performed on an Illumina MiSeq system. Raw data were processed using DADA2 scripts in R platform and quality-filtered reads (~50,862 reads per sample) were used to identify amplicon sequence variants (ASV) by closed reference picking against the Silva database (54). Abundance of selected bacterial strains were confirmed by qPCR using 25 ng of fecal bacterial DNA and specific 16S rRNA primers for *Mucispirillum schaedleri* (ASF457)*, Akkermansia muciniphila, Bifidobacterium*, *Bacteroides* sp. (ASF519) (primer sequences are listed in **Supplemental Table**). qPCRs were performed in duplicate using SsoAdvanced Universal SYBR® Green Supermix. Relative abundance for each strain was quantified by normalizing the quantity of each specific 16S rRNA gene to the total amount of 16S bacterial DNA. 16S rRNA sequences generated in this study are publicly available. This data can be found here: ENA, accession number PRJEB50182 (https://www.ebi.ac.uk/ena/browser/home).

### Culture of Intestinal Enteroids

Enteroid cultures were performed using ileal crypts isolated from WT, *Batf3^-/-^*, and *Irf8^-/-^* mice as described previously (55). Briefly, ileum was collected, washed in ice-cold PBS without Ca^2+^ and Mg^2+^, cut longitudinally, luminal content was washed out with ice-cold PBS, and tissues were cut into 2-3 mm pieces. Pieces were washed in PBS with intermittent mixing to remove villi. Next, tissue pieces were agitated in PBS containing 2 mM EDTA for 20 min at room temperature. Next, tissue pieces were placed in PBS containing 0.1% BSA, gently vortexed for 30-60 s, and filtered through a 70 µm cell strainer to remove tissue pieces. This step was repeated for a total of three times and fractions 2 and 3 were collected and pooled. The filtrates containing crypts were centrifuged, washed with PBS containing 0.1% BSA, and resuspended in Advanced Dulbecco’s modified eagle medium (DMEM)/F12 medium. Matrigel^®^ (Corning, 50% vol/vol) was added to the crypts and seeded into 48-well plates. After polymerization of Matrigel^®^, Intesticult organoid growth media (Stem cell Technologies) was added. Medium was replaced every 2-3 days. Images were acquired using Zeiss Axio Observer.Z1 inverted microscope on days 1, 4, and 6. Sixty to 100 enteroids were used for quantification of enteroid surface area and *de novo* bud formation on day 6. Enteroid surface area was calculated using ZEN2 software. For enteroid formation potential, live, intact, and viable enteroids were counted on day 6 and normalized to counted enteroids on day 1 of the same well. On day 6, RNA was extracted by using RNeasy Micro kit according to the manufacturer’s instructions (Qiagen).

### Quantification of IgA^+^ Coated Fecal Bacteria

Fecal pellets were collected from 8-week-old WT and *Batf3^-/-^* mice and reconstituted in 1 ml PBS containing 1% BSA (w/v) overnight at 4°C. Samples were processed as described (27). In brief, fecal bacteria were used for IgA staining and cell-free supernatants of fecal matter were used for IgA ELISA. Secreted IgA concentrations were normalized to the total weight of fecal pellets. For flow cytometry analysis of IgA-coated bacteria, bacterial suspensions were stained with PE-anti-IgA (eBioscience) or corresponding isotype control followed by staining with Sytox Green. Samples were acquired by flow cytometry using an LSR II analyzer (BD Biosciences) and analyzed using FlowJo software (TreeStar Inc.). The following gating strategy was used: bacteria were gated based on forward and side scatter excluding cell aggregates followed by gating on Sytox Green^+^ IgA^low^ or Sytox Green^+^ IgA^high^.

### Statistics

The data are represented as mean ± standard error of the mean (SEM), or standard deviation (SD) as indicated with the number of mice (n) specified in the figure legends. Data were pooled from at least two independent experiments. The unpaired two-tailed Student *t*-test or Mann-Whitney-U test was applied as indicated. Differences were considered significant at *p* < 0.05. For microbiome analysis, alpha diversity metrics included Faith’s phylogenetic diversity (Faith’s PD) metric, Chao1, and Shannon index. The significance of differences in alpha diversity was calculated by *t*-tests and non-parametric Wilcoxon signed rank tests. Beta diversity was calculated using square root Jensen-Shannon divergence and visualized by principal coordinates analysis. Association of microbial genera with *Batf3^-/-^* mice were evaluated using DESeq2 in R, which employs an empirical Bayesian approach to shrink dispersion and fit non-rarified count data to a negative binomial model (56). *P*-values for differential abundance were converted to q-values to correct for multiple hypothesis testing (< 0.05 for significance) (57).

## Results

### *Batf3^-/-^* Mice Develop Metabolic Syndrome

**Figure 1:**
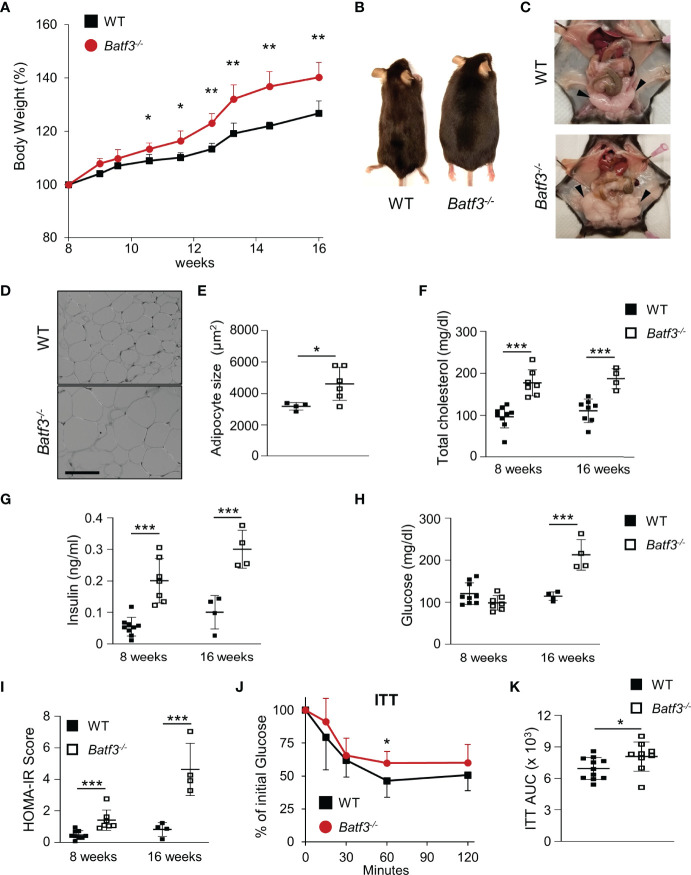
*Batf3^-/-^* mice develop metabolic syndrome. **(A)** Body weight gain as percentage of the initial weight at 8 weeks (n = 4/group). **(B, C)** Representative images of WT and *Batf3^-/-^* mice **(B)** and abdominal cavity **(C)** indicating gonadal white adipose tissue (GWAT; black arrow heads) at 6 months of age. **(D)** Representative H&E staining of GWAT at 16 weeks of age (Scale bar, 100 μm). **(E)** Quantification of adipocyte sizes (100 adipocytes/mouse; n = 4-6/genotype). **(F)** Serum total cholesterol concentrations after overnight fasting (8 weeks, n = 7-9/genotype; 16 weeks, n = 4-8/genotype). **(G–I)** Serum insulin concentrations **(G)**, fasting blood glucose concentrations **(H)**, and homeostatic model assessment for insulin resistance (HOMA-IR) score **(I)** at 8 and 16 weeks of age (8 weeks, n = 7-9/genotype; 16 weeks, n = 4-8/genotype). **(J, K)** Insulin tolerance test (ITT) **(J)** and area under the curve (AUC) analysis of ITT **(K)** in WT and *Batf3^-/-^* mice at 16 weeks of age (n = 9-11/genotype). Data are represented as means ± SD. Statistical significance was determined by Student’s *t*-test. *p < 0.05, **p < 0.01, ***p < 0.005.

To evaluate the role of BATF3 in the development of metabolic syndrome, we first administered standard chow and measured the percentage of weight gain of WT and *Batf3^-/-^* mice from the age of 8 to 16 weeks (**Figure S1A**). We observed that *Batf3^-/-^* mice had greater weight gain over time compared to WT mice (**Figures 1A, B**). The difference in weight gain between WT and *Batf3^-/-^* mice was significant as early as 11 weeks of age and observed in both female and male mice (**Figure S1B**). Interestingly, the increased body weight in *Batf3^-/-^* mice was not associated with increased food intake (**Figure S1C**). Furthermore, we observed an increase in the size of abdominal fat deposits and a trend toward greater weight of gonadal fat tissue but no change in the weight of livers in *Batf3^-/-^* compared to WT mice (**Figures 1C**, **S1D, E**). Increased body weight in *Batf3^-/-^* mice was associated with increased abdominal white adipocyte size (**Figures 1D, E**), and spontaneous development of hepatosteatosis (**Figures S1E, F**). In addition to the development of obesity in *Batf3^-/-^* mice, we also observed symptoms of metabolic syndrome in *Batf3^-/-^* mice. We observed an increase in serum total cholesterol (**Figure 1F**), and fasting insulin levels (**Figure 1G**) in *Batf3^-/-^* mice as early as 8 weeks of age, which preceded the development of obesity, and increased fasting blood glucose levels in *Batf3^-/-^* mice compared to WT mice at 16 weeks of age (**Figure 1H**). Next, we determined the homeostatic model assessment for insulin resistance (HOMA-IR) score, which takes into consideration the fasting blood glucose and serum insulin concentrations and gives a score for early insulin resistance (HOMA-IR > 1.9, early insulin resistance; > 2.9, significant insulin resistance) (46). At 8 weeks of age *Batf3^-/-^* mice had a significantly higher HOMA-IR score than WT mice but it did not reach the threshold for insulin resistance of 1.9 (**Figure 1I**). However, at 16 weeks of age *Batf3^-/-^* mice had a HOMA-IR score of 4.6, while WT mice had a HOMA-IR score of 0.8, indicating insulin resistance in *Batf3^-/-^* mice (**Figure 1I**). To confirm our findings, we performed insulin tolerance tests in 8- and 16-week-old mice. *Batf3^-/-^* mice had significantly higher blood glucose concentration after i.p. injection of insulin compared to WT mice at 16 weeks, indicating insulin resistance in these mice (**Figures 1J, K**). These findings suggest that an early increase in total cholesterol and serum insulin concentration in *Batf3^-/-^* mice might contribute to the development of insulin resistance and metabolic syndrome.

### BATF3-Deficiency Leads to a Loss of Intestinal Barrier Integrity

**Figure 2:**
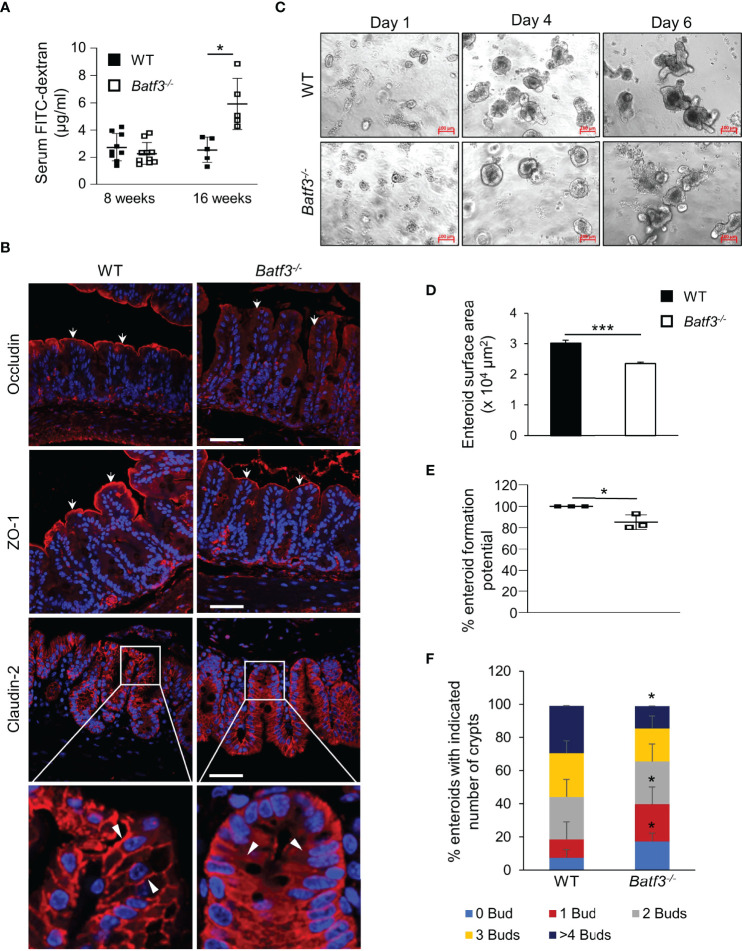
BATF3-deficiency leads to a loss of intestinal barrier integrity. **(A)** Intestinal permeability assay measuring serum concentration of FITC-labeled dextran in WT and *Batf3^-/-^* mice 3 h post FITC-dextran gavage at 8 and 16 weeks of age (8 weeks, n = 9-10/genotype; 16 weeks, n = 5/genotype). **(B)** Representative cecal immunofluorescence images of Occludin, ZO-1, Claudin-2 (all red), counterstained with DAPI (blue) at 16 weeks of age (Scale bar, 50 μm). White arrows indicate apical expression of Occludin, and ZO-1 on the intestinal epithelial layer. White arrow heads indicate membrane expression of Claudin-2 in WT (left panel), and cytosolic expression in *Batf3***^-/-^** mice (right panel). **(C)** Representative phase contrast images of the ileal enteroids developed from WT and *Batf3***^-/-^** mice captured on days 1, 4, and 6 using inverted microscope. Three independent experiments were performed (n=3/group) (Scale bar, 100 µm). **(D–F)** Characterization of enteroids on day 6 for **(D)** enteroid surface area, **(E)** enteroid formation potential, and **(F)** *de novo* crypt formation (budding). Three independent experiments were performed (n = 60-100 enteroids/genotype/experiments). Data are represented as means ± SD **(A)** or ± SEM **(D, E)**. Statistical significance was determined by unpaired *t*-test (for analyzing enteroid surface area, and % enteroid formation potential) or by Mann-Whitney-U test (for *de novo* crypt formation, and FITC-dextran assay). *p < 0.05, ***p < 0.005.

Leaky gut has been associated with inflammatory bowel diseases (IBD) and development of metabolic syndrome. Several studies have suggested that loss of the intestinal barrier could lead to translocation of bacteria and an increase of bacterial metabolites in the blood (16). To assess the role of BATF3 in maintaining intestinal barrier, we quantified intestinal permeability *in vivo* by gavaging WT and *Batf3^-/-^* mice with FITC-dextran and measuring FITC-dextran levels in the serum (**Figure 2A**). At 16 weeks of age, *Batf3^-/-^* mice had significantly higher serum FITC-dextran concentrations than WT mice, indicating that intestinal permeability was significantly increased in *Batf3^-/-^* mice. However, at 8 weeks of age, *Batf3^-/-^* and WT mice had similar serum FITC-dextran concentrations (**Figure 2A**). Tight junctions are multiprotein complexes that regulate intestinal permeability. Several studies suggest that obesity and hyperglycemia could lead to alterations of tight junctions (20, 22, 58). To examine if such alterations occurred in *Batf3^-/-^* mice, we analyzed the localization of several tight junction proteins by immunofluorescence staining of cecal sections from 16-week-old *Batf3^-/-^* and WT mice (**Figure 2B**). Interestingly, the tight junction protein Occludin is localized mainly at the cell membrane in WT mice, however, in *Batf3^-/-^* mice, Occludin is localized predominantly in the cytoplasm. Furthermore, the membrane expression of ZO-1 is reduced in *Batf3^-/-^* mice compared to WT mice. Finally, Claudin-2 is localized at the membrane surface and the cytoplasm in *Batf3^-/-^* mice while it is only present at the cell surface in WT mice (**Figure 2B**). Next, we assessed the regenerative potential of intestinal epithelial cells *ex vivo* using intestinal enteroid cultures (59). Crypts were isolated from the ileum of 4-month-old WT and *Batf3^-/-^* mice and cultured for 6 days. We observed a smaller size of the spherical crypts (spheroids) on days 1 and 4 in *Batf3^-/-^* mice compared to WT mice (**Figure 2C**). Furthermore, surface area was significantly reduced for enteroids developed from *Batf3^-/-^* mice compared to WT mice (**Figure 2D**) suggesting regenerative defects. Moreover, we observed that the enteroid formation potential was significantly reduced in *Batf3^-/-^* compared to WT enteroids (**Figure 2E**). Lastly, enteroids that developed from *Batf3^-/-^* mice have significantly fewer numbers of crypts per enteroid (**Figure S2A**) and the percentage of enteroids with zero or one bud were significantly higher compared to enteroids from WT mice (**Figure 2F**). Conversely, the percentages of enteroids with four or more buds were significantly higher in WT compared to *Batf3^-/-^* mice (**Figure 2F**). Collectively, these data indicate regenerative defects in *ex vivo* enteroid cultures from *Batf3^-/-^* mice. To determine if these defects might be due to a cell-intrinsic role of BATF3 in intestinal epithelial cells (IEC), we measured *Batf3* mRNA expression of whole ileal tissue, isolated primary IEC, and enteroids from WT and *Batf3^-/-^* mice. *Batf3* was undetectable in IEC or enteroids developed from WT or *Batf3^-/-^* mice at steady state, while we were able to detect *Batf3* in whole ileal tissue from WT but not *Batf3^-/-^* mice (**Figures S2B, C**). Muc2 was readily expressed in WT and *Batf3^-/-^* primary IEC demonstrating the successful isolation of IEC. To assess if enteroids developed from *Batf3^-/-^* mice had similar defects in tight-junction proteins compared to cecal tissues from *Batf3^-/-^* mice *in vivo*, we used immunofluorescent stainings for tight junction proteins ZO-1, Occludin, and Claudin-2 and quantified fluorescent intensities in enteroids. Fluorescent intensity was significantly reduced for ZO-1 and Occuldin in enteroids from *Batf3^-/-^* mice compared to WT enteroids, while Claudin-2 fluorescent intensities are similar in WT and *Batf3^-/-^* mice (**Figures S2D, E**). To better understand the role of cDC1 in maintaining intestinal homeostasis, we used mice deficient in the transcription factor IRF8 which interacts with BATF3 in cDC1 development. Consistent with previous publications, *Irf8^-/-^* mice lack lamina propria cDC1 similar to *Batf3^-/-^* mice (**Figure S2F**) (60). We observed that IRF8 was also expressed in whole ileal tissue, but in contrast to BATF3, IRF8 was expressed in intestinal organoids, and isolated IEC (**Figure S2G**). However, lack of IRF8 did not affect enteroid surface area, enteroid formation potential, or the numbers of crypts per enteroid compared to enteroids derived from WT mice (**Figures S2H-J**). Furthermore, percentages of buds per crypt were similar between enteroids from *Irf8^-/-^* mice and WT mice (**Figure S2K**). Taken together, our results demonstrate an important role for BATF3 in maintaining intestinal homeostasis and suggests that this role is independent of its expression in epithelial cells at least under homeostatic conditions.

### BATF3-Deficiency Leads to a Shift Toward a Pro-Inflammatory Phenotype in the Lamina Propria

**Figure 3:**
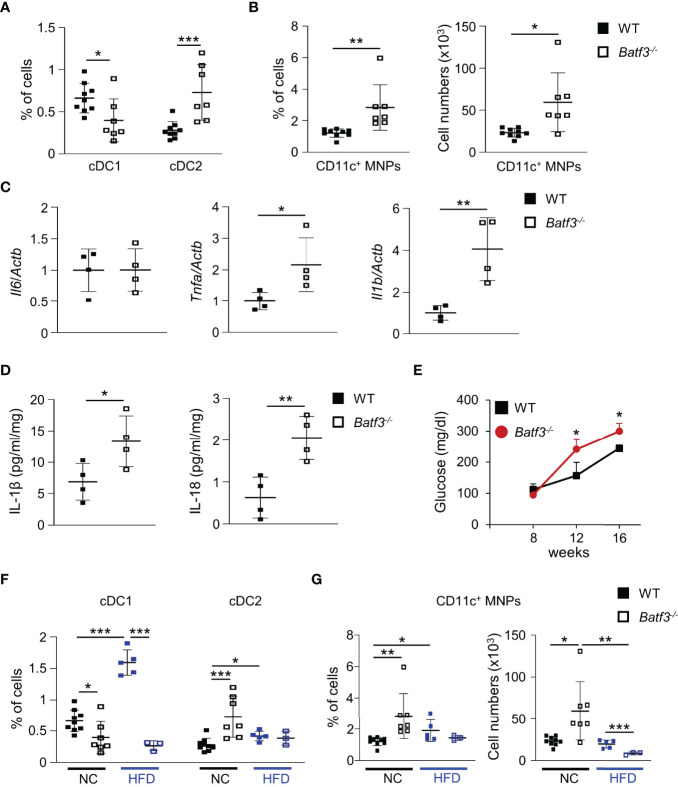
BATF3-deficiency leads to a shift toward a pro-inflammatory phenotype in the lamina propria. **(A, B)** Quantification of colonic lamina propria cDC1, and cDC2 **(A)**, and CD11c^+^ MNPs, percentage of cells (left panel), total number of cells per large intestine (right panel) **(B)** of WT and *Batf3***^-/-^** mice at 16 weeks (WT, n = 9; *Batf3***^-/-^**, n = 7). **(C)** mRNA expression of indicated genes in the cecum of 16-week-old mice measured by qPCR. All data were normalized to expression of *Actb* and represented as fold changes compared to WT mice (n = 4/group). **(D)** IL-1β and IL-18 secretion from colon explants of WT and *Batf3***^-/-^** mice at 16 weeks measured by ELISA (n = 4/group). **(E–G)** WT and *Batf3^-/-^* mice were fed a HFD from 8 to 16 weeks of age. **(E)** Fasting blood glucose levels during HFD. **(F, G)** Quantification of cDC1, and cDC2 **(F)**, and CD11c^+^ MNPs, percentage of cells (left panel), total number of cells per large intestine (right panel) **(G)** of 16-week-old WT and *Batf3***^-/-^** mice on normal chow or HFD (NC: n = 7-9/group; HFD: n = 3-5/group). Data are represented as means ± SD. Statistical significance was determined by Student’s *t*-test. *p < 0.05, **p < 0.01, ***p < 0.005.

Next, we characterized the innate immune cells composition within the large intestinal lamina propria (LP) of WT and *Batf3^-/-^* mice at 8 and 16 weeks of age. At 8 weeks of age, we observed an almost complete loss of cDC1, as expected, but no significant changes in the percentages of cDC2 or pro-inflammatory CD11c^+^ MNPs in *Batf3^-/-^* compared to WT mice (**Figures S3A, B**). Lack of cDC1 was not associated with spontaneous intestinal inflammation as shown by H&E stainings of the cecum of WT and *Batf3^-/-^* mice at 8 and 16 weeks of age (**Figures S3C, D**). However, at 16 weeks we observed reduction of cDC1, and a concomitant significant increase in the percentages of cDC2 and CD11c^+^ MNPs in *Batf3^-/-^* mice compared to WT mice in the LP (**Figures 3A, B**, **S3E, F**). The partial recovery of cDC1 abundance in 16-week-old *Batf3^-/-^* mice might be due to the inflammatory milieu in the LP as has been shown during infections in *Batf3^-/-^* mice (61). As an increase of CD11c^+^ MNPs is often associated with low-grade inflammation, we next analyzed the expression level of pro-inflammatory cytokines by qPCR in cecal tissue. *Batf3^-/-^* mice had increased expression of the pro-inflammatory cytokines *Tnfa* and *Il1b*, but not *Il6* (**Figure 3C**). Next, we analyzed secretion of IL-1β and IL-18 in *ex-vivo* colonic explants. *Batf3^-/-^* explants had a significant increase in secretion of IL-1β and IL-18 compared to WT explants (**Figure 3D**). Taken together, these findings demonstrate that the lack of BATF3 leads to an increase in pro-inflammatory innate immune cells and increased expression of pro-inflammatory cytokines in the large intestinal LP.

### High-Fat Diet Exacerbates DSS-Induced Acute Colitis in *Batf3^-/-^* Mice

**Figure 4:**
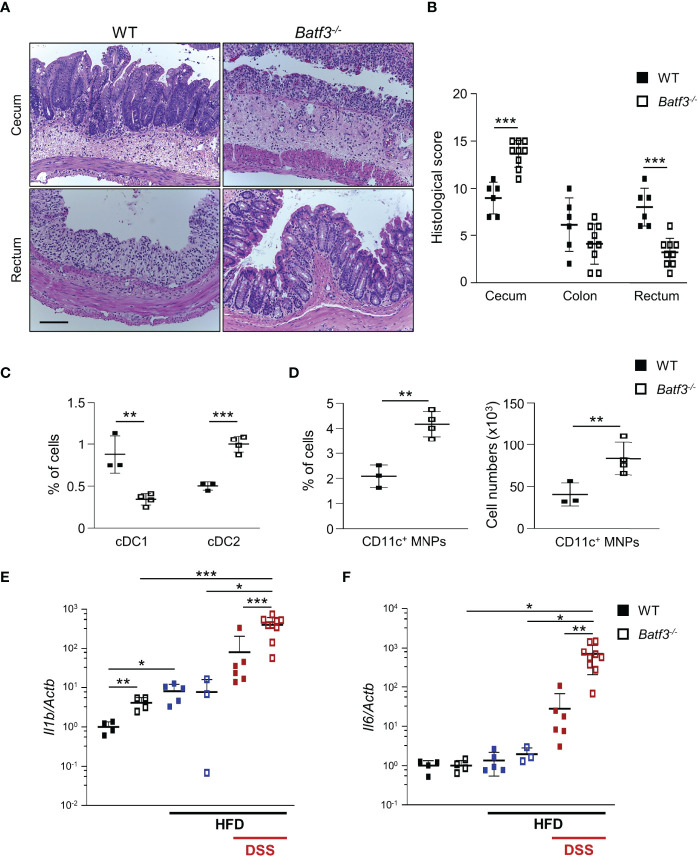
High-fat diet exacerbates DSS-induced acute colitis in *Batf3^-/-^* mice. **(A)** Representative H&E staining of cecum and rectum from WT and *Batf3^-/-^* mice receiving high fat diet (HFD) for 8 weeks followed by 7 days of DSS drinking water (Scale bar, 100 μm). **(B)** Histological scores for cecum, colon, and rectum (n = 6-9/genotype). **(C, D)** Percentages of lamina propria cDC1, cDC2 **(C)**, and CD11c^+^ MNPs, percentage of cells (left panel), total number of cells per large intestine (right panel) **(D)** measured by flow cytometry (n = 3-4/genotype). One representative experiment out of two independent experiments is shown. **(E, F)** mRNA expression of *Il1b* **(E)** and *Il6* **(F)** in the cecum of 16-week-old mice on normal chow, receiving HFD, and receiving HFD and 7 days of DSS as measured by qPCR. All data were normalized to expression of *Actb* and represented as fold changes compared to WT mice (NC: n = 4/genotype; HFD: n = 3-5/genotype; HFD + DSS: n = 6-9/genotype). Data are represented as means ± SD. Statistical significance was determined by Student’s *t*-test. *, *p* < 0.05, **, *p* < 0.01, ***, *p* < 0.005.

Next, we determined if BATF3-deficiency also impacts diet-induced obesity. We administered a high-fat diet (HFD, 60% of total calories from fat) to WT and *Batf3^-/-^* mice from 8 to 16 weeks of age (**Figure S4A**). HFD resulted in comparable total body weights at 16 weeks in WT and *Batf3^-/-^* mice (**Figure S4B**). However, we observed an increased fasting glucose, increased liver weight, and hepatosteatosis in *Batf3^-/-^* mice (**Figures 3E**, **S4C, D**). In contrast to normal chow, the size of white adipocytes was not significantly different between WT and *Batf3^-/-^* mice on HFD (**Figures S4E, F**). Interestingly, HFD feeding led to a significant increase in the percentage of cDC1, and CD11c^+^ MNPs in WT, but a decrease of CD11c^+^ MNPs in *Batf3^-/-^* mice compared to normal chow (**Figures 3F, G**). Furthermore, we observed significantly reduced percentage of cDC1 in *Batf3^-/-^* mice on normal chow, as expected, and on HFD, but did not observe any differences in cDC2 between *Batf3^-/-^* mice and WT mice on HFD (**Figure 3F**). To elucidate the impact that metabolic syndrome and barrier dysfunction in *Batf3^-/-^* mice might have on susceptibility to DSS colitis, we administered an HFD to WT and *Batf3^-/-^* mice from 8 to 16 weeks of age followed by 7 days of DSS drinking water (**Figures S5A**). Acute DSS colitis in mice that were fed a HFD did not lead to significant differences in body weight, but resulted in more proximal inflammation in *Batf3^-/-^* compared to WT mice with rectal sparing, a phenotype that is reminiscent of human Crohn’s disease (**Figures 4A, B**, **S5B**). *Batf3^-/-^* mice have significantly higher histological scores in the cecum and lower scores in the rectum compared to WT mice (**Figure 4B**). Similar to what we observed under normal chow, *Batf3^-/-^* mice have an increased percentage of cDC2 and CD11c^+^ MNPs compared to WT mice in the LP (**Figures 4C, D**), increased levels of IL-1β and IL-6 (**Figures 4E, F**), and increased size of white adipocytes (**Figures S5C, D**). TNFα is not significantly different between WT and *Batf3^-/-^* mice under HFD with acute DSS colitis (**Figure S5E**). HFD alone also increased the expression of IL-1β and TNFα in WT and *Batf3^-/-^* mice, respectively (**Figures 4E**, **S5E**). However, we only observed significant differences in the expression of TNFα, but neither IL-1β, nor IL-6 between WT and *Batf3^-/-^* mice on HFD alone (**Figures 4E, F**, **S5E**). These data support our hypothesis, that metabolic syndrome and the shift to a pro-inflammatory LP phenotype observed in *Batf3^-/-^* mice contribute to the increased susceptibility of injury induced colitis in *Batf3^-/-^* mice.

### The Development of Metabolic Syndrome in *Batf3^-/-^* Mice Is Dependent on Intestinal Microbiota

**Figure 5:**
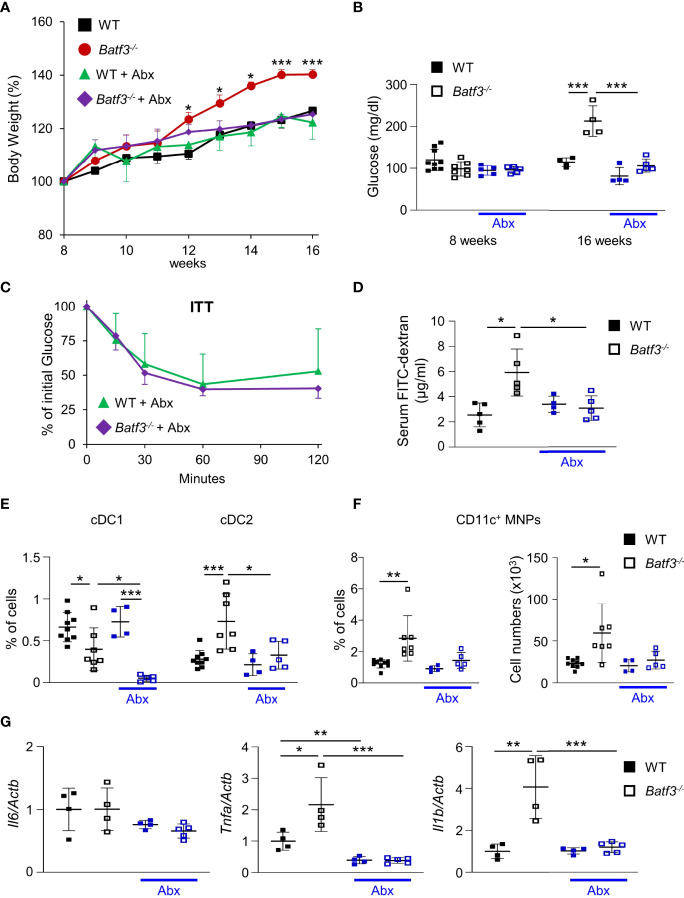
Commensal bacteria contribute to the development of metabolic syndrome in *Batf3^-/-^* mice. **(A)** Body weight gain as percentage of the initial weight at week 8 (NC: n = 4/genotype; Abx: n = 4-5/genotype). **(B)** Fasting blood glucose concentrations (NC: n = 4/genotype; Abx: n = 4-5/genotype). **(C)** Insulin tolerance test (ITT) in WT and *Batf3^-/-^* mice at 16 weeks of age treated with Abx (NC: n = 4/genotype; Abx: n = 4-5/genotype). **(D)** FITC-dextran serum concentration at 16 weeks of age (NC: n = 4/genotype; Abx: n = 4-5/genotype). **(E, F)** Percentage of colonic lamina propria cDC1, cDC2 **(E)**, and CD11c^+^ MNPs, percentage of cells (left panel), total number of cells per large intestine (right panel) **(F)** measured by flow cytometry (NC: n = 4/genotype; Abx: n = 4-5/genotype). **(G)** mRNA expression of *Il1b*, *Tnfa*, and *Il6* in the cecum of 16-week-old mice on normal chow, with or without Abx as measured by qPCR. All data were normalized to expression of *Actb* and represented as fold changes compared to WT mice (NC: n = 4/genotype; Abx: n = 4-5/genotype). Data are represented as means ± SD. Statistical significance was determined by Student’s *t*-test (A-C, E-G), or Mann-Whitney-U test **(D)**. *p < 0.05, **p < 0.01, ***p < 0.005.

The gut microbiota are a known contributor to metabolic function and has been linked to the development of human metabolic syndrome (12, 62). The interplay between intestinal microbiota and immune responses has been shown to be critical in preventing dysbiosis and subsequently metabolic syndrome. To investigate the role of intestinal bacteria in the development of metabolic syndrome in *Batf3^-/-^*, we treated WT and *Batf3^-/-^* mice with broad-spectrum antibiotics (Abx) from the time of weaning until 16 weeks of age (**Figure S6A**). WT mice showed no differences in weight gain with Abx treatment (**Figure 5A**). In contrast, in *Batf3^-/-^* mice treated with Abx, weight gain was completely reduced to weight gain seen in WT mice treated with or without Abx (**Figure 5A**). This was accompanied by a reduction of fasting glucose, insulin, and total cholesterol levels in *Batf3^-/-^* mice to WT levels (**Figures 5B**, **S6B, C**). Furthermore, insulin tolerance of *Batf3^-/-^* mice treated with Abx was similar to WT mice treated with Abx (**Figure 5C**). Treatment with Abx also restored impaired intestinal permeability of *Batf3^-/-^* mice to WT level as measured by serum FITC-dextran (**Figure 5D**). Furthermore, treatment with Abx significantly reduced the percentage of LP cDC2 and CD11c^+^ MNPs in *Batf3^-/-^* mice to percentages comparable to WT mice (**Figures 5E, F**). The reduced percentages of cDC2 and CD11c^+^ MNPs in Abx treated *Batf3^-/-^* mice was accompanied by a significant reduction in expression of IL-1β, and TNFα in the cecum of *Batf3^-/-^* mice (**Figure 5G**). In summary, these data show an important role of the intestinal microbiota in the development of metabolic syndrome and pro-inflammatory LP phenotype observed in *Batf3^-/-^* mice.

### BATF3-Deficiency Leads to Altered IgA-Coating of Bacteria and Intestinal Dysbiosis Preceding the Development of Metabolic Syndrome

**Figure 6:**
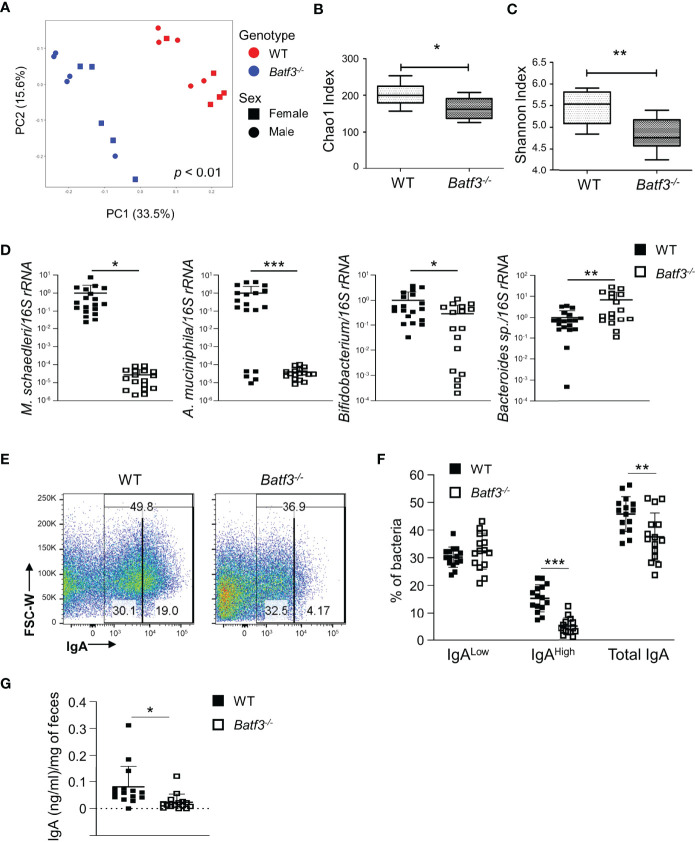
BATF3-deficiency leads to intestinal dysbiosis preceding the development of metabolic syndrome. **(A)** Principal Coordinates Analysis (PCoA) of fecal microbiota of 8-week-old WT and *Batf3^-/-^* mice fed standard chow with consideration for genotype and sex of mice (n = 10/genotype). **(B, C)** Chao1 index **(B)** and Shannon index **(C)** of fecal microbiota of 8-week-old WT and *Batf3^-/-^* mice (n = 10/genotype). **(D)** Quantification of relative abundance of *Mucispirillum schaedleri, Akkermansia muciniphila, Bifidobacterium*, *Bacteroides* sp. in fecal samples of 8-week-old WT and *Batf3^-/-^* mice by qPCR (n = 17-19/genotype). **(E)** Representative flow cytometry plots of IgA coated fecal bacteria isolated from WT and *Batf3^-/-^* mice. **(F)** Quantification of IgA^low^, IgA^high^ and total IgA coated bacteria in feces of WT and *Batf3***^-/-^** mice at 8 weeks of age (n =15/genotype). **(G)** Secreted IgA from fecal samples of WT and *Batf3***^-/-^** mice at 8 weeks of age measured by ELISA (n = 15/genotype). Data are represented as means ± SD **(A, D, F, G)** or median (bar), interquartile range (box), and range (whisker) **(B, C)**. Statistical significance was determined by Adonis test **(A)**, or Student’s *t*-test **(B–D, F, G)**. *p < 0.05, **p < 0.01, ***p < 0.005.

To further investigate the role of intestinal microbiota in the development of metabolic syndrome in *Batf3^-/-^* mice, we performed 16S rRNA gene sequencing on 8-week-old WT and *Batf3^-/-^* mice before the onset of metabolic syndrome. We observed significantly altered microbiome composition in fecal samples of *Batf3^-/-^* compared to WT mice using principal coordinates analysis (PCoA) with significant separation of WT and *Batf3^-/-^* clusters independently of the sexes of the mice (**Figure 6A**). Furthermore, alpha diversity was significantly reduced in *Batf3^-/-^* compared to WT mice as analyzed by Chao1 and Shannon alpha diversity indices (**Figures 6B, C**). Decreased intestinal bacterial diversity has been associated with increased inflammation and obesity (29, 30, 63). At the phylum level, *Batf3^-/-^* mice had a lower abundance of *Firmicutes*, *Verrucomicrobia*, *Actinobacteria*, and a higher abundance of *Bacteroidetes* compared to WT mice (**Figure S7A**). At the genus level, *Batf3^-/-^* mice have a higher abundance of *Prevotellacea*, *Bacteroides*, *Lactobacillus*, and a lower abundance of *Akkermansia* and *Bifidobacterium* (**Figure S7B**). Next, we used a negative binomial model to identify differentially abundant microbial genera. *Akkermansia muciniphila*, *Mucispirillum schaedleri*, *Angelakisella*, and *Bifidobacterium* were most differentially abundant with a high abundance in WT mice while *Parabacteroides*, *Prevotellaceae*, and *Lactobacillus* were more abundant in *Batf3^-/-^* mice (**Figure S7C**). The abundance of *A. muciniphila*, a mucolytic bacterial species, has been negatively correlated with obesity and metabolic syndrome in animal models and humans (64–68). We confirmed our 16S rRNA data in a larger cohort by qPCR. At 8 weeks of age *Batf3^-/-^* mice had a significantly increased abundance of *Bacteroides* sp. and decreased abundance of *A. muciniphila*, *M. schaedleri*, and *Bifidobacterium* (**Figure 6D**). We next investigated mechanisms that could contribute to microbial dysbiosis. We isolated intestinal fecal contents from WT and *Batf3^-/-^* mice to determine the percentage of total IgA-coated, IgA^high/low^-coated bacteria, and fecal secretory IgA (sIgA). *Batf3^-/-^* mice had a significantly lower percentage of total IgA and IgA^high^-coated bacteria compared to WT mice, while the percentage of IgA^low^-coated bacteria was similar (**Figures 6E, F**, **S7D**). Furthermore, fecal secreted IgA was significantly reduced in *Batf3^-/-^* mice compared to WT mice (**Figure 6G**). Our data suggest that BATF3-deficiency leads to altered IgA-coating of bacteria, and intestinal dysbiosis that could contribute to the development of metabolic syndrome.

### Treatment With 2-DG Reverses the Inflammatory Phenotype and Impaired Barrier Function in *Batf3^-/-^* Mice

**Figure 7:**
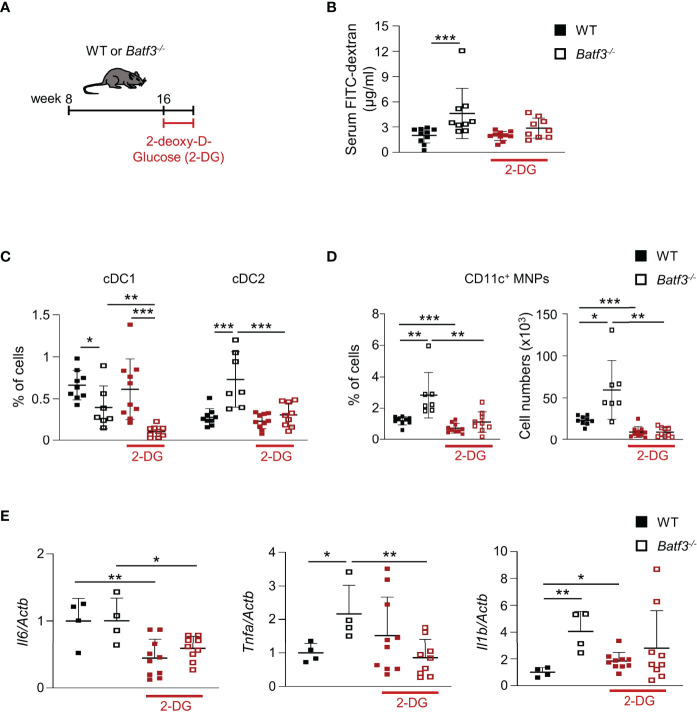
Treatment with 2-DG restores intestinal barrier integrity and reverses the inflammatory phenotype in *Batf3^-/-^* mice. **(A)** Schematic of 2-DG treatment of 16-week-old WT and *Batf3^-/-^* mice. **(B)** FITC-dextran serum concentration of WT and *Batf3^-/-^* mice before and after treatment with 2-DG (n = 9-10/genotype). **(C, D)** Percentages of lamina propria cDC1, cDC2 **(C)**, and CD11c^+^ MNPs, percentage of cells (left panel), total number of cells per large intestine (right panel) **(D)** measured by flow cytometry (n = 9-10/genotype). **(E)** mRNA expression of *Il6, Tnfa*, and *Il1b* in the cecum of mice treated with 2-DG for 10 days as measured by qPCR. All data were normalized to expression of *Actb* and represented as fold changes compared to WT mice (n = 9-10/genotype). Data are represented as means ± SD. Statistical significance was determined by Student’s *t*-test **(C–E)**, or Mann-Whitney-U test **(B)**. *p < 0.05, **p < 0.01, ***p < 0.005.

To assess whether the hyperglycemia that we observed in *Batf3^-/-^* mice contributes to the impaired intestinal barrier and subsequent pro-inflammatory phenotype in *Batf3^-/-^* mice, we treated 16-week-old WT and *Batf3^-/-^* mice with 2-DG, an inhibitor of glucose metabolism, for 10 days (**Figure 7A**). Treatment with 2-DG reduced serum concentration of FITC-Dextran in *Batf3^-/-^* mice to levels similar of WT mice (**Figure 7B**), suggesting an improvement in intestinal epithelial barrier function. Moreover, treatment with 2-DG restored the percentage of cDC2 and CD11c^+^ MNPs of *Batf3^-/-^* mice to levels seen in WT mice without a major impact on cDC1 (**Figures 7C, D**). Treatment of *Batf3^-/-^* mice with 2-DG significantly reduced the expression level of TNFα and IL-6 in *Batf3^-/-^* mice comparable to WT expression levels (**Figure 7E**). These findings suggest a key role of epithelial glucose metabolism in the impairment of the intestinal barrier in *Batf3^-/-^* mice contributing to the development of metabolic syndrome in these mice.

## Discussion

The intestinal immune system and its interaction with intestinal microbiota has emerged as an important player in the development of metabolic syndrome (12, 16, 69). Multiple factors contributing to the development of metabolic syndrome converge at the site of the intestinal barrier: absorption of nutrients from diet, gut microbiota, and the intestinal immune system (17, 69). While changes in the composition of the intestinal immune system under high fat diets have been observed and associated to the development of an impaired intestinal barrier, the exact mechanisms of specific intestinal DC subsets contributing to metabolic changes that lead to a leaky barrier and associated low-grade inflammation and metabolic syndrome are largely unknown. Here, we identified the transcription factor BATF3 as an important contributor to the complex interactions between mucosal cDCs, intestinal microbiota, epithelial cells, host immune responses, and metabolism. Our study identified BATF3 to be important in orchestrating intestinal epithelial barrier function and an anti-inflammatory milieu during homeostasis. BATF3-deficiency leads to early changes in IgA-coating of bacteria, intestinal microbiota composition, hyperinsulinemia, and hypercholesterolemia under normal caloric intake. The metabolic changes lead progressively to the development of obesity, hyperglycemia, and metabolic syndrome. Hyperglycemia fragilized the large intestinal epithelial barrier by disturbing the localization of several tight-junction proteins leading to increased intestinal permeability. We also observed a significant shift in the population of lamina propria DC subsets and macrophages toward a pro-inflammatory phenotype leading to low-grade inflammation. Previous observations of BATF3-dependent cDC1s being most abundant in the colon is consistent with our findings of impaired colonic permeability, tight junction alterations, and pro-inflammatory milieu (70). Our findings demonstrate that BATF3 is required to maintain a healthy intestinal barrier and prevent the development of intestinal dysbiosis and chronic low-grade inflammation that contributes to the development of metabolic syndrome.

The earliest metabolic changes that we observed in *Batf3^-/-^* mice were increased serum insulin and total cholesterol, which preceded impaired intestinal barrier function, obesity, and changes in lamina propria immune composition. Serum lipids and free fatty acids can induce insulin resistance and impaired glucose metabolism, and can also activate inflammatory pathways in innate immune cells *via* TLR4 recognition of these lipids (71). At later time-points, we observed impaired intestinal barrier integrity and a concomitant shift toward a pro-inflammatory milieu in the lamina propria that included an increase in CD11c^+^ MNPs. While adipose tissue M1 macrophages are associated with obesity and insulin resistance, the overall numbers of small intestinal macrophages are increased in human obesity but their contribution to disease development and progression is less clear (72, 73). Moreover, a previous study investigating the role of DCs in obesity and insulin resistance used CD11c depletion strategy, which depletes the majority of intestinal cDC subsets with different functions (74). Using *Batf3^-/-^* mice with a specific depletion of lamina propria CD103^+^ cDC1 suggests a role for this subset in the development of metabolic syndrome and the pro-inflammatory phenotype.

Hyperglycemia has been demonstrated to markedly interfere with homeostatic intestinal epithelial barrier function by altering the expression and localization of adherence and tight junction proteins (22). We demonstrate that *Batf3^-/-^* mice present with impaired intestinal barrier function at 16 weeks of age, which is associated with the development of insulin resistance and hepatosteatosis. Mechanistically, treatment of obese *Batf3^-/-^* mice with the glycolysis inhibitor 2-DG for 10 days restored intestinal barrier integrity, significantly decreased the low-grade inflammation, and reversed the shift in DC subsets and macrophages in the lamina propria of these mice. Although the treatment with 2-DG for 10 days may not be sufficient to reverse the metabolic syndrome in these mice, it demonstrates the central role of glucose metabolism in intestinal epithelial cells to the loss of barrier integrity and pro-inflammatory phenotype in the gut of *Batf3^-/-^* mice. 2-DG is a non-specific glycolysis inhibitor and treatment will impact many biological processes in addition to its effect on IEC. A previous publication has demonstrated that hyperglycemia can directly impact IEC function *in vitro* and *in vivo* and that 2-DG blocks this effect *in vivo*. Our data demonstrate a decrease in epithelial barrier permeability and a shift of inflammatory DC/Macrophages in *Batf3^-/-^* mice toward a WT phenotype, but there may be other contributing factors in our model.

Alteration in intestinal microbiota have been associated with the development of obesity and metabolic syndrome (25). Several mechanisms have been proposed for how microbiota induce the development of obesity and metabolic syndrome, including digestion of nutrients, storage of body fat, and efficiency of energy harvesting from the diet (23–25). Obesity-associated dysbiosis of microbiota has also been linked to increased permeability and gut inflammation (18–20). Alterations in IgA-coating of luminal bacteria are associated with changes of microbial composition leading to the development of obesity (27, 75). The sensing of dysbiosis and bacterial products by innate immune cells *via* TLRs and down-stream signaling pathways will trigger chronic inflammation and metabolic syndrome. Although several TLRs and down-stream signaling molecules including MyD88, and NLRPs have been implicated in the development of obesity, these proteins are broadly expressed on intestinal DCs, macrophages, and T cells (15, 30, 76–78). However, the role of specific lamina propria cell types that potentially lead to and/or detect dysbiosis, and breach of the intestinal barrier in the context of obesity are not clearly defined. Here, we show that *Batf3^-/-^* mice display microbial dysbiosis characterized by an increased abundance of *Bacteroides* sp. and decreased abundance of *A. muciniphila*, *M. schaedleri*, and *Bifidobacterium*. The abundance of *A. muciniphila* is inversely correlated with obesity, type 2 diabetes, and metabolic syndrome, and supplementation with *A. muciniphila* improved several metabolic parameters in obese human volunteers (65, 79–81). In HFD-induced obesity in mice, oral administration of *A. muciniphila* could reverse metabolic syndrome including fat-mass gain, metabolic endotoxemia, adipose tissue inflammation, and insulin resistance without influencing food intake (65, 82). Moreover, *A. muciniphila*-derived extracellular vesicles could regulate the expression of tight-junction proteins and decrease intestinal permeability *in vivo* and in Caco-2 cells (83, 84). Protective effects against devolvement of obesity have been attributed to *Bifidobacterium animalis* ssp. in mouse models of HFD-induced obesity by the production of short chain fatty acids and their effects on adipocyte metabolism and host energy expenditure (85–87). *Lachnospiraceae* and their metabolites, short chain fatty acids, also mitigate HFD-induced obesity, insulin resistance, and intestinal inflammation (30). Interestingly, while a direct link to metabolic function has not been established for *M. schaedleri*, colonization protects against salmonella infection by competing for anaerobic respiration substrates and down-regulating the type 3 secretion system of *S. enterica* serovar Typhimurium (88). Taken together, these studies and our findings suggest that changes in the abundance of specific bacteria in *Batf3^-/-^* mice may trigger or contribute to the development of metabolic syndrome. This hypothesis is supported by our data, demonstrating that antibiotic treatment of *Batf3^-/-^* mice prevented the development of metabolic syndrome and low-grade inflammation. Thus, BATF3 plays an important role in preventing the development of metabolic syndrome through a microbiome-dependent mechanism.

Previous studies using *Batf3^-/-^* mice did not observe any spontaneous development of intestinal inflammation or increased susceptibly to DSS colitis which is consistent with our findings in 8- and 16-week-old *Batf3^-/-^* mice (43) (**Figures S3C, D**). However, we hypothesized that in the context of obesity, BATF3-deficiency may increase susceptibly to acute DSS colitis. To exclude any confounding effects of obesity on its own, we administrated HFD to WT and *Batf3^-/-^* mice. On HFD, *Batf3^-/-^* mice had a higher susceptibly to cecal inflammation during acute DSS colitis compared to WT mice. Our data suggest that BATF3 plays a protective role in the development of intestinal inflammation under conditions of metabolic syndrome. Interestingly, a sizable subgroup of patients with IBD are obese and visceral adiposity has been associated with an increased risk of IBD-related complications and poor response to medical therapy suggesting an integrated relationship between obesity and the pathogenesis of IBD (89).

A recent study demonstrated that BATF3 is expressed by intestinal epithelial cells in a model of colitis-associated colon cancer and in human cancer cell lines (90). We did not detect *Batf3* mRNA expression in *ex vivo* cultures of enteroids generated from WT mice which is consistent with a previous publication (90). Our data suggest, that in WT mice BATF3 was mainly expressed by lamina propria immune cells rather than IEC under steady state conditions. Although enteroids did not express BATF3, enteroid formation potential was significantly reduced in *Batf3^-/-^* mice compared to WT mice, which could be explained by either cell-intrinsic defects of tight junction proteins or the cell-extrinsic microenvironment imprinting on the regenerative potential of *Batf3^-/-^* enteroids. Previous publications have demonstrated that enteroids derived from patients with IBD recapitulated histological and functional features of the primary tissues, including the absence of acidic mucus secretion and aberrant adherens junctions in the epithelial barrier by mechanisms involving epigenetic modifications such as DNA methylation in intestinal stem cells (91–93). We observed changes in the expression and localization of tight junction proteins ZO-1 and Occludin in enteroids derived from *Batf3^-/-^* mice that recapitulate our *in vivo* findings.

BATF3-dependent DCs are mainly present as tissue–resident and migratory DCs characterized as CD103^+^CD11b^−^ in the lamina propria while in lymphoid organs BATF3-dependent DCs express CD8α (43). Although our study focused on the consequences of deficiency in BATF3-dependent cDC1s in the lamina propria, a role of BATF3-dependent DCs in other organs that contribute to metabolic syndrome can’t be excluded. *Batf3^-/-^* mice have been recently shown to be more susceptible to the development of steatohepatitis after administration of high sucrose diet by mechanisms that include BATF3-dependent hepatic cDC1 regulation of inflammatory cell influx and lipid metabolism (94). Furthermore, BATF3 expression has been reported in other cell types, including regulatory T cells, CD4^+^ T_H_9, and CD8^+^ T cells (95–98). In addition to *Batf3^-/-^* mice, we also examined *Irf8^-/-^* mice as a genetic model lacking cDC1. *Irf8^-/-^* mice did not develop metabolic syndrome and we did not observe any deficiency in enteroid formation, most likely because the defects in enteroid formations in *Batf3^-/-^* mice are driven by metabolic changes including hyperglycemia. The additional lack of monocytes/macrophages in *Irf8^-/-^* mice most likely contributes to a different microbiota compared to *Batf3^-/-^* mice which only lack cDC1.

A recent study investigated the role of BATF3 and cDC1s in the regulation of adipose tissue homeostasis (99). The authors demonstrate that the abundance of cDC1s in visceral fat tissue is reduced in HFD fed mice and that BATF3-deficiency and the loss of cDC1 results in the development of obesity during ageing. Mechanistically, BATF3-deficiency caused adipose tissue inflammation characterized by an increase in M1-like adipose tissue macrophages and TNFα expression and a decrease of invariant NKT (iNKT) cells that precedes the development of obesity. Treatment with FLT3L, which leads to the expansion of cDC1 but also to a lesser degree of cDC2 in adipose tissue, reduced weight gain, hyperlipidemia, and increased the abundance of iNKT cells. Depletion of iNKT cells in the context of FLT3L treatment resulted in a loss of the protective effects. These data suggest, that the cDC1-iNKT cell axis controls adipose tissue homeostasis.

In summary, we demonstrate that BATF3 plays a protective role in the development of metabolic syndrome by mechanisms involving the intestinal microbiome, regulation of intestinal epithelial cell homeostasis, and prevention of low-grade intestinal inflammation.

## Data Availability Statement

The original contributions presented in the study are publicly available. This data can be found here: ENA, PRJEB50182.

## Ethics Statement

The animal study was reviewed and approved by Cedars-Sinai Medical Center Animal Care and Use Committee.

## Author Contributions

Conceptualization, HH and KM; Methodology, HH and KM; Investigation, HH, JS, DS, SM, LT, AB, NL, SC, BS, YS, and HG; Writing – Original Draft, HH and KM; Writing – Review & Editing, HH, ST, and KM; Funding Acquisition, ST and KM. All authors contributed to the article and approved the submitted version.

## Funding

This work was supported by the F. Widjaja Foundation (ST and KM) and the UCSD/UCLA DRC Pilot grant to KM (P30 DK063491). DS received a Student Research Award by the Crohn’s and Colitis Foundation of America. NL received funding from Sorbonne University Paris (Master Integrative Biology and Physiology), Direction des Relations Internationales and from the “CROUS”.

## Conflict of Interest

The authors declare that the research was conducted in the absence of any commercial or financial relationships that could be construed as a potential conflict of interest.

## Publisher’s Note

All claims expressed in this article are solely those of the authors and do not necessarily represent those of their affiliated organizations, or those of the publisher, the editors and the reviewers. Any product that may be evaluated in this article, or claim that may be made by its manufacturer, is not guaranteed or endorsed by the publisher.
