# Supplementary material for: BATF3 Protects Against Metabolic Syndrome and Maintains Intestinal Epithelial Homeostasis
Source: Front Immunol. 2022 Jun 22;13:841065. doi: 10.3389/fimmu.2022.841065 (PMC9257242; doi:10.3389/fimmu.2022.841065)
Supplement: Supplementary file 1 [file Table_1.pdf]

Supplemental Table

Primer and Taqman probe sequences		
Mouse Il6	Thermo Fisher Scientific	Cat# 4331182; Assay ID Mm99999064_m1
Mouse Tnfa	This paper	Forward 5'-TTC TCA AAA TTC GAG TGA CAA GCC-3'<br>Reverse 5'-CTG GGA GTA GAC AAG GTA CAA CC-3'<br>Probe 5'-FAM-AGC CCA CGT CGT AGC AAA CCA CCA-BHQ-3'
Mouse Il1b	This paper	Forward 5'-CAC TAC AGG CTC CGA GAT GAA C-3'<br>Reverse 5'-ATT TTG TCG TTG CTT GGT TCT CC-3'<br>Probe 5'-FAM-AAA GCC TCG TGC TGT CGG ACC CAT-BHQ-3'
Mouse Actb	This paper	Forward 5'-ATG ACC CAG ATC ATG TTT GA-3'<br>Reverse 5'-TAC GAC CAG AGG CAT ACA G-3'<br>Probe 5'-FAM-CGT AGC CAT CCA GGC TGT-BHQ-3'
Mouse Batf3	This paper	Forward 5'- TGC AGA GAA GCG TGG ATG-3'<br>Reverse 5'- CTC TCC TTC GAA CTT TCC TGT C-3'<br>Probe 5'-FAM- CGC AGA GCC CCA AGG ACG A-3'
Mouse Muc2	This paper	Forward 5'- CCC TGA AGA AGA ACC CAC CTG-3'<br>Reverse 5'- CAG TCA AAC TCA AAG TGC TCT CC-3'
Mouse Irf8	This paper	Forward 5'- GAT GAG AAG ACC ATG TTC CG-3'<br>Reverse 5'- AGC ATC CAC CTC CTG ATT-3'
16S rRNA universal primers	(1)	Forward 5'- ACTCCTACGGGAGGCAGCAGT-3'<br>Reverse 5'- ATTACCGCGGCTGCTGGC-3'
Akkermansia muciniphila	(2)	Forward 5'-CAGCACGTGAAGGTGGGGAC-3'<br>Reverse 5'- CCTTGCGGTTGGCTTCAGAT-3'
ASF457 (*Mucispirillum schaedleri*)	(3)	Forward 5'- CCGAAAGGTGAGCTAATGCCGG-3'<br>Reverse 5'- GGGACGCGAGTCCATCTTTC-3'
Bifidobacterium spp.	(4)	Forward 5'- GCGTGCTTAACACATGCAAGTC-3'<br>Reverse 5'- CACCCGTTTCCAGGAGCTATT-3'
ASF519 (*Bacteroides* sp.)	(3)	Forward 5'- CACAGTAAGCGGCACAGCG-3'<br>Reverse 5'- CCGCTCACACGGTAGCTG-3'

1. J.H. Skalski, J.J. Limon, P. Sharma, M.D. Gargus, C. Nguyen, J. Tang, A.L. Coelho, C.M. Hogaboam, T.R. Crother, and D.M. Underhill, Expansion of commensal fungus Wallemia mellicola in the gastrointestinal mycobiota enhances the severity of allergic airway disease in mice. PLoS Pathog 14 (2018) e1007260.
2. M.C. Collado, M. Derrien, E. Isolauri, W.M. de Vos, and S. Salminen, Intestinal integrity and Akkermansia muciniphila, a mucin-degrading member of the intestinal microbiota present in infants, adults, and the elderly. Appl Environ Microbiol 73 (2007) 7767-70.

3. R.B. Sarma-Rupavtarm, Z. Ge, D.B. Schauer, J.G. Fox, and M.F. Polz, Spatial distribution and stability of the eight microbial species of the altered schaedler flora in the mouse gastrointestinal tract. Appl Environ Microbiol 70 (2004) 2791-800.
4. J. Penders, C. Vink, C. Driessen, N. London, C. Thijs, and E.E. Stobberingh, Quantification of Bifidobacterium spp., Escherichia coli and Clostridium difficile in faecal samples of breast-fed and formula-fed infants by real-time PCR. FEMS Microbiol Lett 243 (2005) 141-7.
