# Supplementary material for: BATF3 Protects Against Metabolic Syndrome and Maintains Intestinal Epithelial Homeostasis
Source: Front Immunol. 2022 Jun 22;13:841065. doi: 10.3389/fimmu.2022.841065 (PMC9257242; doi:10.3389/fimmu.2022.841065)
Supplement: Supplementary file 2 [file DataSheet_1.pdf]

Figure S1

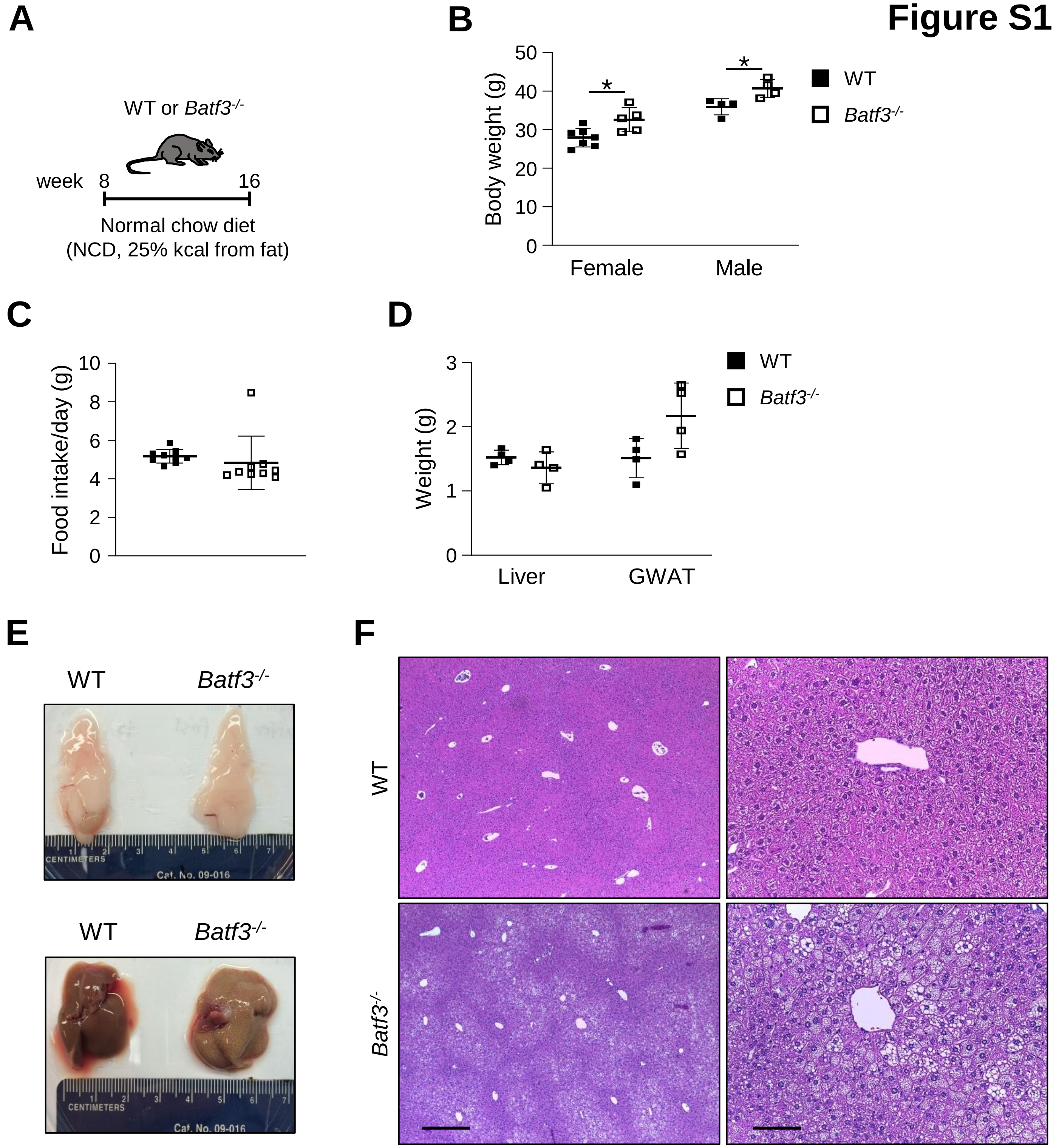

A
WT or Batf3-/-
week 8 16
Normal chow diet
(NCD, 25% kcal from fat)
B
Body weight (g)
50
40
30
20
10
0
*
*
Female
Male
WT
Batf3-/-
C
Food intake/day (g)
10
8
6
4
2
0
D
Weight (g)
3
2
1
0
Liver
GWAT
WT
Batf3-/-
E
WT
Batf3-/-
WT
Batf3-/-
F
WT
Batf3-/-

Figure S1: *Batf3$^{-/-}$* mice develop metabolic syndrome and hepatosteatosis. (A) Experimental setup for WT and *Batf3$^{-/-}$* mice receiving standard chow from the age of 8 to 16 weeks. **(B)** Body weights of WT and *Batf3$^{-/-}$* mice at 16 weeks of age (Female: n = 4-6/group; Male: n = 4/group). **(C)** Food intake in WT and *Batf3$^{-/-}$* mice per day (n = 9-10/group). **(D-F)** Organ weights **(D)**, representative images of liver and gonadal white adipose tissue (GWAT) **(E)**, and representative H&E staining images of liver from WT and *Batf3$^{-/-}$* mice at 16 weeks of age (**F**) (n = 4/group). (Left panels, scale bar 400 μm; right panels, scale bar 100 μm). Data are represented as means ± SD. Statistical significance was determined by Student's *t*-test. *, $p < 0.05$.

Figure S2

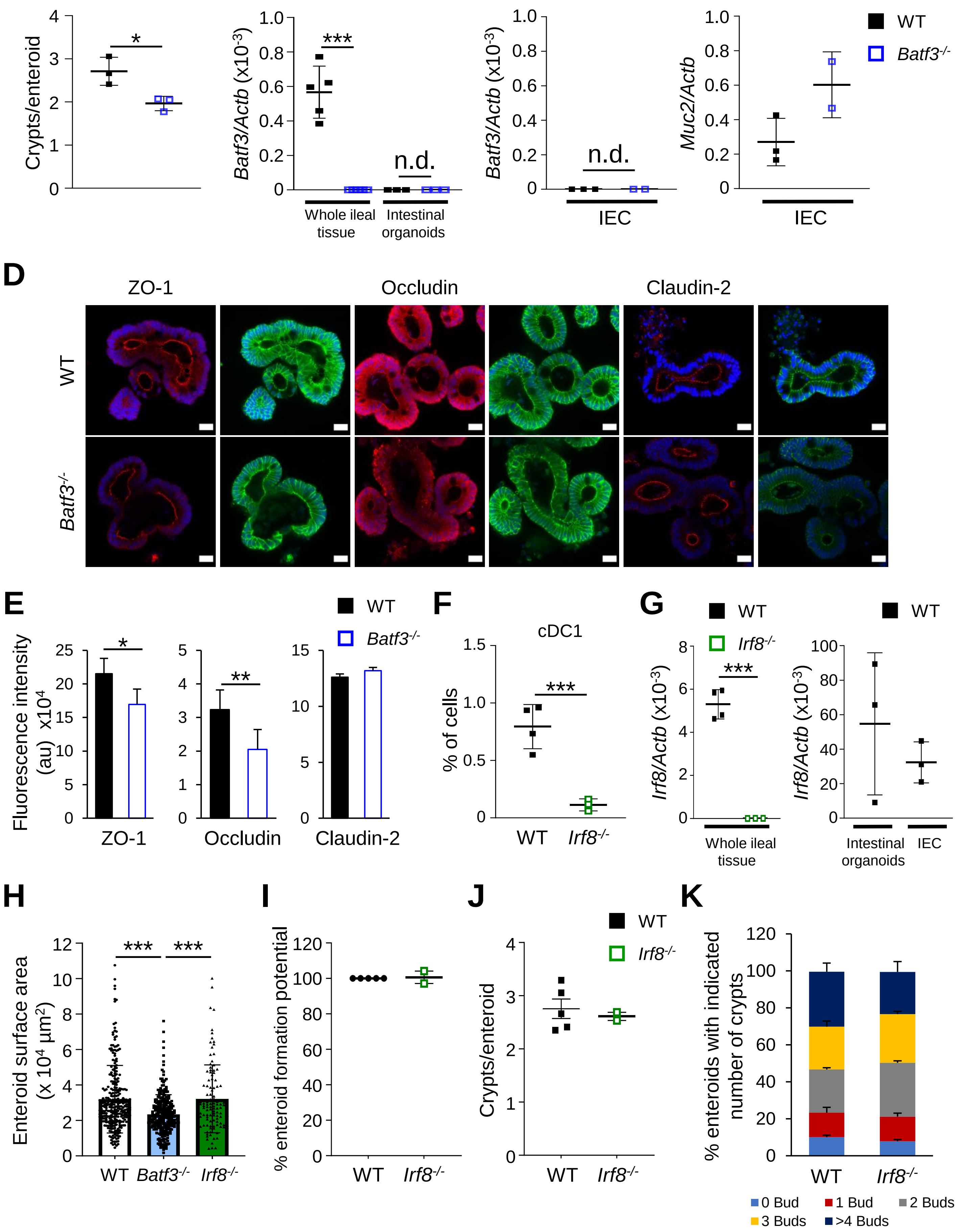

A
Crypts/enteroid
*
B
Batf3/Actb (x10-3)
***
n.d.
Whole ileal tissue
Intestinal organoids
C
Batf3/Actb (x10-3)
n.d.
IEC
Muc2/Actb
IEC
WT
Batf3-/-
D
ZO-1
Occludin
Claudin-2
WT
Batf3-/-
E
Fluorescence intensity (au) x10^4
*
**
ZO-1
Occludin
Claudin-2
WT
Batf3-/-
F
cDC1
% of cells
***
WT
Irf8-/-
G
WT
Irf8-/-
Irf8/Actb (x10-3)
***
Whole ileal tissue
WT
Intestinal organoids
IEC
H
Enteroid surface area (x 10^4 µm^2)
***
***
WT
Batf3-/-
Irf8-/-
I
% enteroid formation potential
WT
Irf8-/-
J
WT
Irf8-/-
Crypts/enteroid
WT
Irf8-/-
K
% enteroids with indicated number of crypts
WT
Irf8-/-
0 Bud
1 Bud
2 Buds
3 Buds
>4 Buds

Figure S2: BATF3- but not IRF8-deficiceny leads to impaired enteroid formation. (A) Characterization of ileal enteroids on day 6. Numbers of crypts per enteroids. Three independent experiments were performed (n = 60-100 enteroids /genotypes/experiment). **(B, C)** mRNA expression of *Batf3* in whole ileal tissue, and intestinal enteroids from WT and *Batf3*$^{-/-}$ mice as measured by qPCR. All data were normalized to expression of *Actb* and represented as relative expression level (n = 5/group for whole ileal tissue; n = 3/group for intestinal enteroids). **(B)** *Batf3*, (**C**) *Batf3* (left) and *Muc2* (right) in primary intestinal epithelial cells (IEC) isolated from small intestines (n = 2-3/group). **(D, E)** Immunofluorescent stainings for tight junction proteins in ileal enteroids. Enteroids derived from WT or *Batf3*$^{-/-}$ mice were stained for ZO-1 (left), Occludin (middle), Claudin-2 (right) (all in red), E-cadherin (green), and counterstained with Hoechst 33342 dye (blue) **(D)**. Quantification of fluorescence intensities for ZO-1, Occludin, and Claudin-2 (n=24-40 enteroids/genotype) **(E)**. **(F)** Quantification of lamina propria cDC1 percentage of cells WT and *Irf8*$^{-/-}$ mice (WT, n = 4; *Irf8*$^{-/-}$, n = 3). (**G**) mRNA expression of *Irf8* in whole ileal tissue from WT and *Irf8*$^{-/-}$ mice (left), intestinal enteroids, and primary IEC isolated from WT mice (right) as measured by qPCR. (**H-K**) Characterization of ileal enteroids from WT, *Batf3*$^{-/-}$, and *Irf8*$^{-/-}$ mice on day 5. (**H**) enteroid surface area, (**I**) enteroid formation potential, (**J**) crypts/enteroid, (**K**) de novo crypt formation. Two independent experiments were performed (n = 2-5 mice/genotype with at least 100 enteroids analyzed per mouse). Data are represented as means ± SD **(B, C, F, G)** or ± SEM (**A, E, H-K**). Statistical significance was determined by Student's *t*-test. *, $p < 0.05$, **, $p < 0.01$, ***, $p < 0.005$.

Figure S3

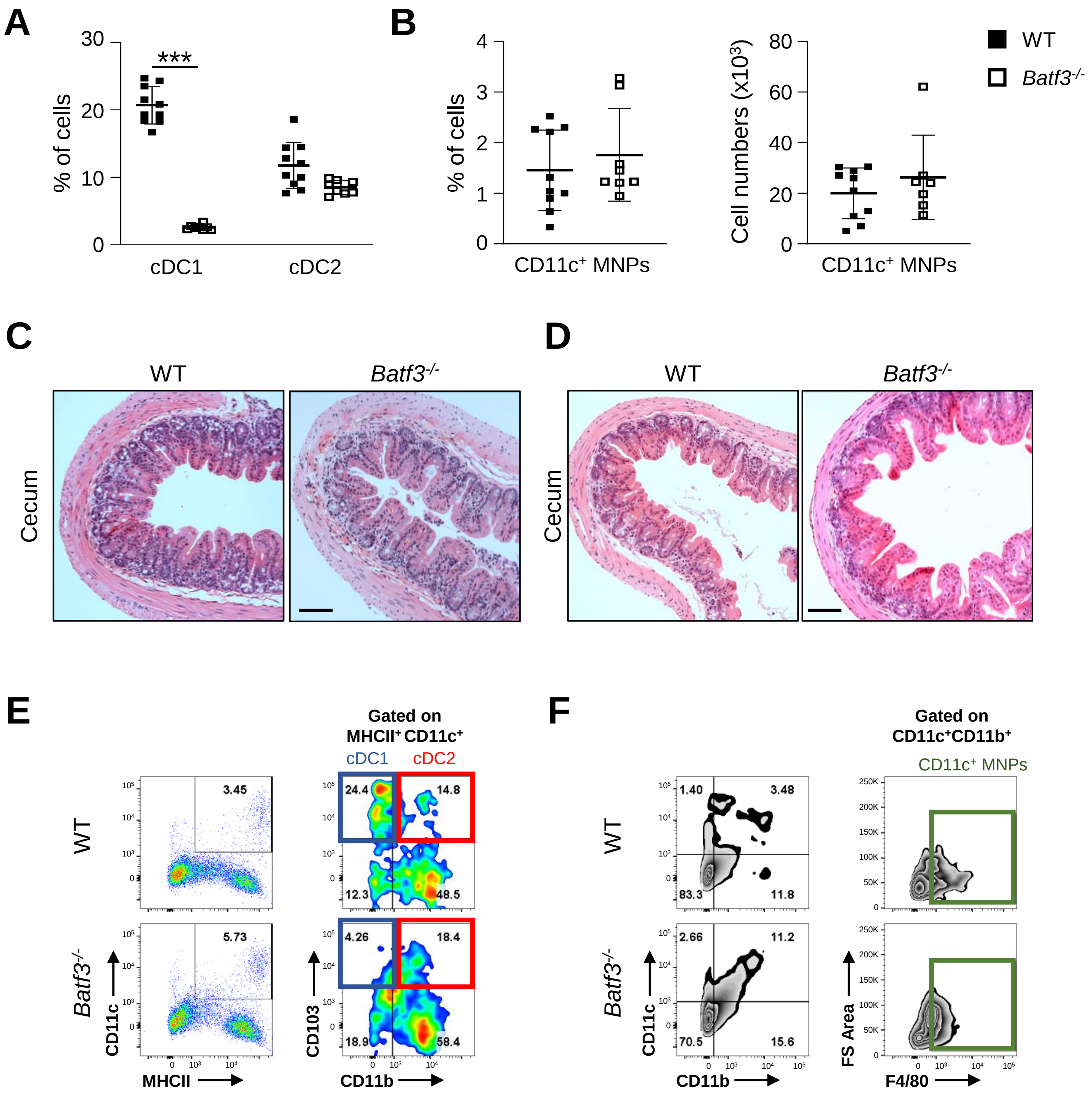

A
% of cells
***
cDC1
cDC2
B
% of cells
CD11c+ MNPs
Cell numbers (x10³)
CD11c+ MNPs
WT
Batf3-/-
C
WT
Batf3-/-
Cecum
D
WT
Batf3-/-
Cecum
E
Gated on
MHCII+ CD11c+
cDC1
cDC2
WT
Batf3-/-
3.45
5.73
24.4
14.8
12.3
48.5
4.26
18.4
18.9
58.4
CD11c
MHCII
CD103
CD11b
F
Gated on
CD11c+CD11b+
CD11c+ MNPs
WT
Batf3-/-
1.40
3.48
83.3
11.8
2.66
11.2
70.5
15.6
CD11c
CD11b
FS Area
F4/80

Figure S3: Kinetics of the shift towards a pro-inflammatory phenotype in the lamina propria in *Batf3*$^{-/-}$ mice. (A, B) Quantification of cDC1, and cDC2 **(A)**, and CD11c^{+} MNPs percentage of cells (left panel), total number of cells per large intestine (right panel) **(B)** of WT and *Batf3*$^{-/-}$ mice at 8 weeks (n = 8-10/group). (**C, D**) Representative H&E stainings of cecum from 8- (**C**) and 16-week-old (**D**) WT and *Batf3*$^{-/-}$ mice. (Scale bar 100 μm). **(E, F)** Gating strategy for flow cytometry analysis of lamina propria cells. LP cells were first gated on FSC-A vs. SSC-A, and single cells were gated on FSC-A vs. FSC-H. Representative flow cytometry plots of cDC1 (MHCII^{+}CD11c^{+}CD103^{+}CD11b^{-}), cDC2 (MHCII^{+}CD11c^{+}CD103^{+} CD11b^{+}) **(E)**, and CD11c^{+} MNPs (CD11b^{+}CD11c^{+}F4/80^{+}) **(F)** from large intestinal lamina propria at 16 weeks of age. Data are represented as means ± SD. Statistical significance was determined by Student's *t*-test. ***, $p < 0.005$.

Figure S4

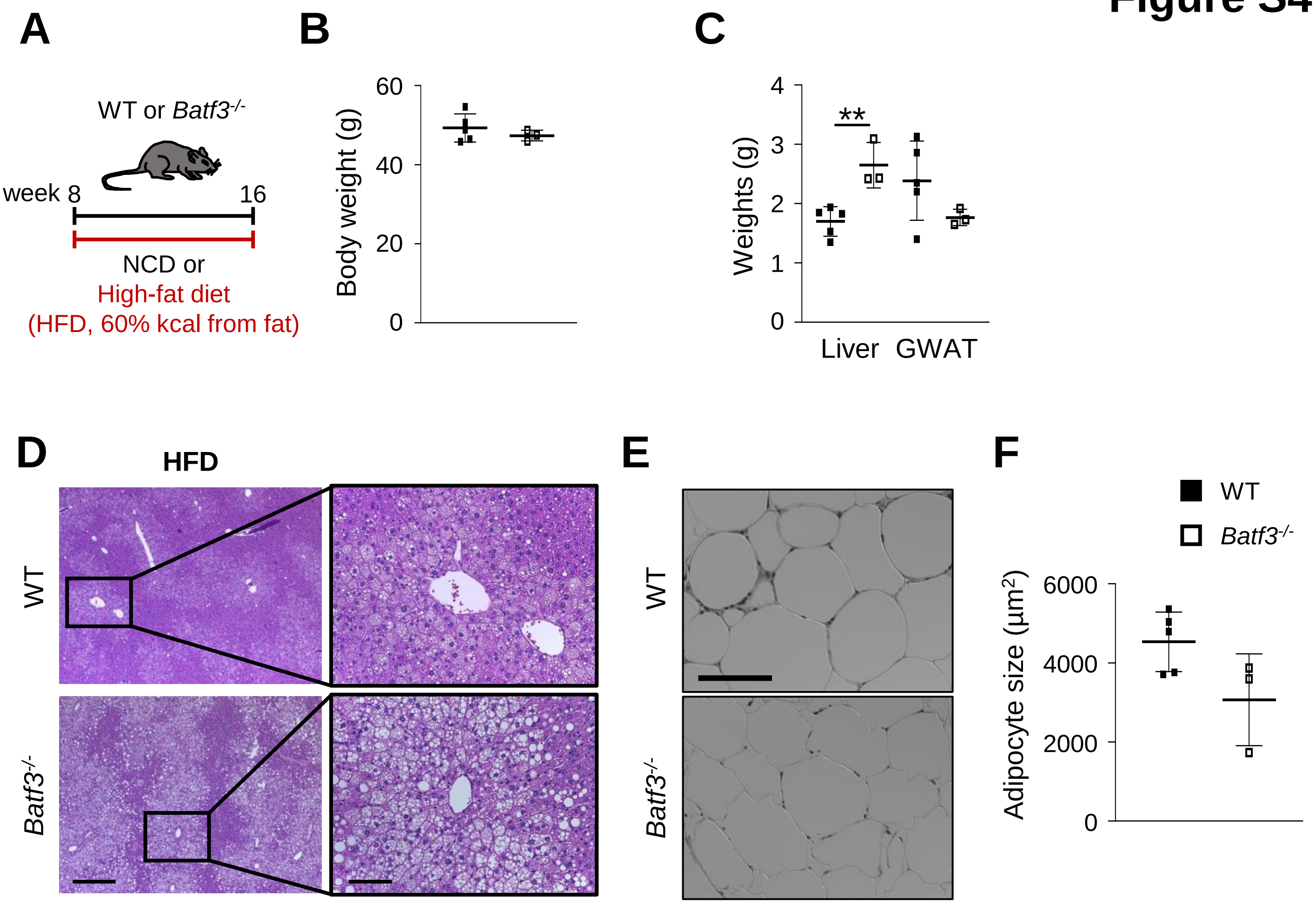

A
WT or Batf3-/-
week 8
16
NCD or
High-fat diet
(HFD, 60% kcal from fat)
B
Body weight (g)
60
40
20
0
C
Weights (g)
**
4
3
2
1
0
Liver
GWAT
D
HFD
WT
Batf3-/-
E
WT
Batf3-/-
F
WT
Batf3-/-
Adipocyte size (μm2)
6000
4000
2000
0

Figure S4: HFD leads to more severe hyperglycemia in *Batf3*$^{-/-}$ mice. (A) Schematic of HFD feeding in WT and *Batf3*$^{-/-}$ mice from 8 to 16 weeks of age. **(B)** Total body weights at 16 weeks (HFD: n = 3-5/group). **(C, D)** Organ weights **(C)**, and representative H&E staining images of liver **(D)** of WT and *Batf3*$^{-/-}$ mice under HFD at 16 weeks (Left panels, scale bar 400 μm; right panels, scale bar 100 μm). **(E, F)** Representative H&E staining of GWAT **(E)**, and quantification of adipocyte sizes **(F)** of WT and *Batf3*$^{-/-}$ mice under HFD at 16 weeks. (Scale bar, 100 μm). Data are represented as means ± SD. Statistical significance was determined by Student's *t*-test. **, $p < 0.01$.

Figure S5

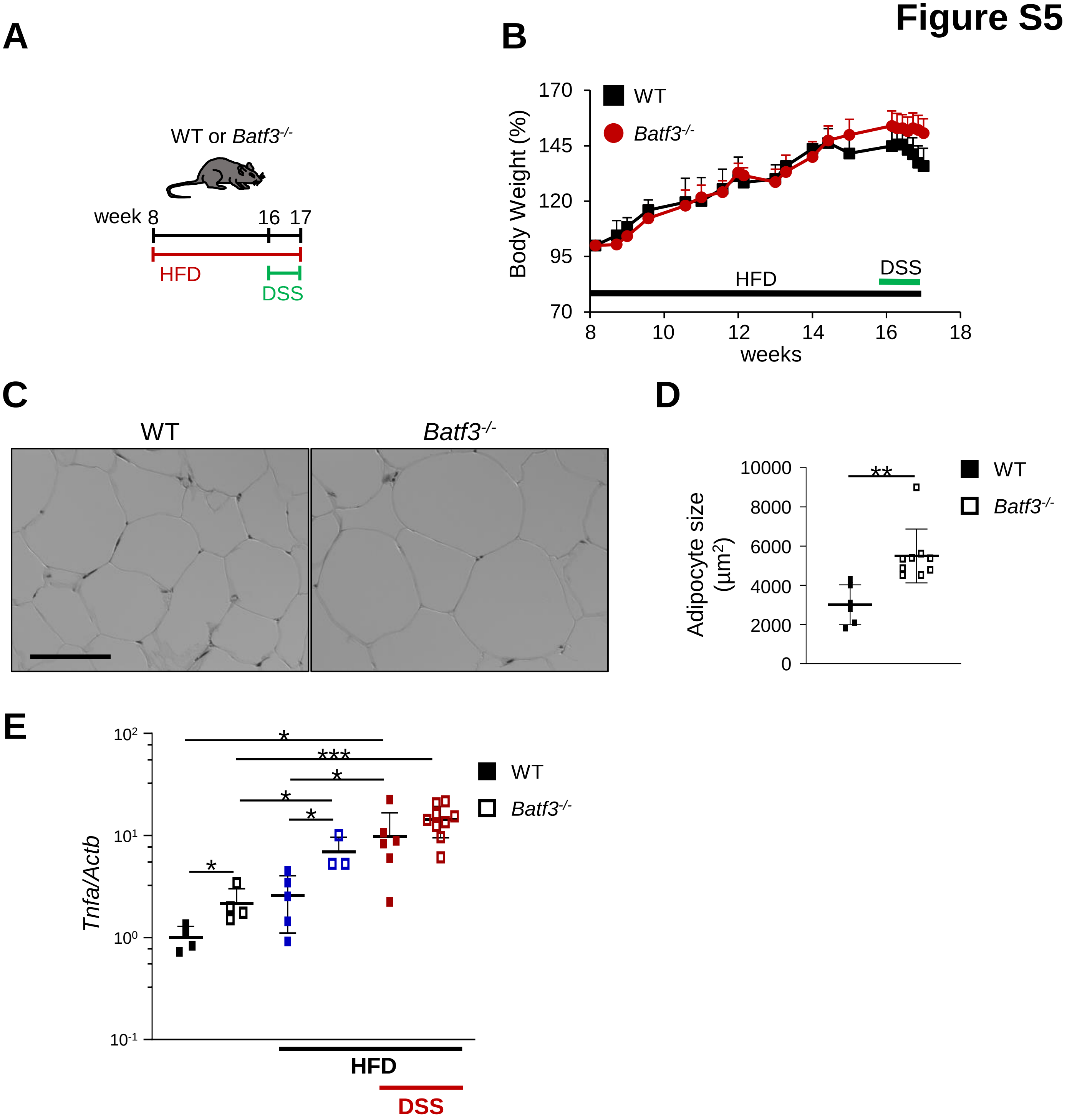

A
WT or Batf3-/-
week 8
16 17
HFD
DSS
B
170
145
120
95
70
Body Weight (%)
WT
Batf3-/-
HFD
DSS
8 10 12 14 16 18
weeks
C
WT
Batf3-/-
D
10000
8000
6000
4000
2000
0
Adipocyte size (µm2)
**
WT
Batf3-/-
E
Tnfa/Actb
102
101
100
10-1
*
***
*
*
*
*
WT
Batf3-/-
HFD
DSS

Figure S5: DSS-induced acute colitis during HFD leads to reduced adipocyte size in WT compared to *Batf3*$^{-/-}$ mice. (A) Schematics of acute DSS-colitis during HFD in WT and *Batf3*$^{-/-}$ mice. **(B)** Body weight gain as percentage of the initial weight at week 8 (n = 6-9/group). **(C, D)** Representative H&E staining of GWAT (**C**), and quantification of adipocyte sizes (**D**) of WT and *Batf3*$^{-/-}$ mice receiving HFD + DSS (Scale bar, 100 μm). **(E)** mRNA expression of *Tnfa* in the cecum of 16-week-old mice on normal chow, receiving HFD, and receiving HFD + DSS as measured by qPCR. All data were normalized to expression of *Actb* and represented as fold changes compared to WT mice (NC: n = 4/group; HFD: n = 3-5/group; HFD + DSS: n = 6-9/group). Data are represented as means ± SD. Statistical significance was determined by Student's *t*-test. *, $p < 0.05$, **, $p < 0.01$, ***, $p < 0.005$.

Figure S6

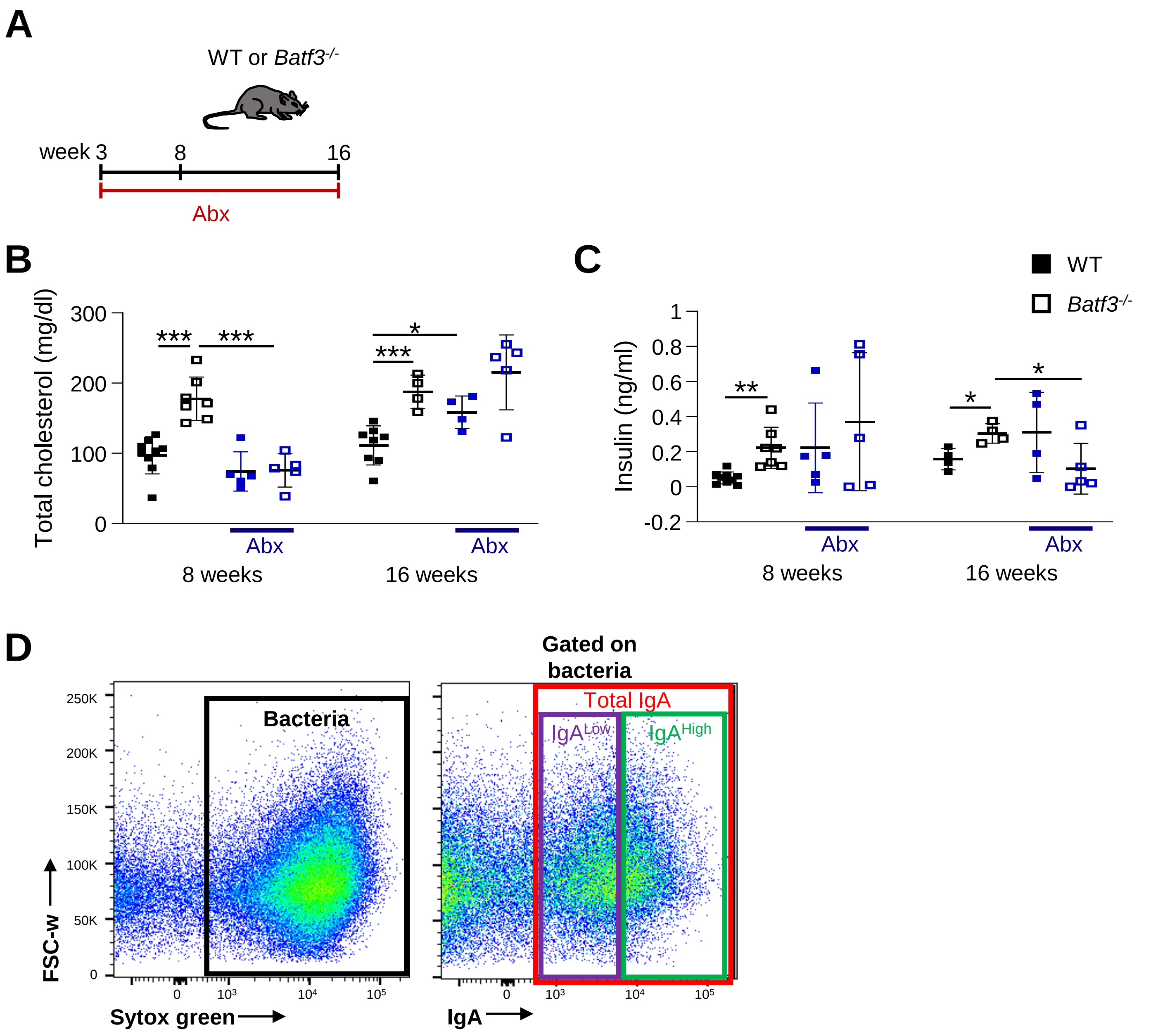

A
WT or Batf3-/-
week 3
8
16
Abx
B
Total cholesterol (mg/dl)
300
200
100
0
***
***
*
***
Abx
Abx
8 weeks
16 weeks
C
WT
Batf3-/-
Insulin (ng/ml)
1
0.8
0.6
0.4
0.2
0
-0.2
**
*
*
Abx
Abx
8 weeks
16 weeks
D
Gated on bacteria
Bacteria
Total IgA
IgA Low
IgA High
FSC-w
250K
200K
150K
100K
50K
0
0
10³
10⁴
10⁵
Sytox green
IgA

Figure S6: Commensal bacteria contribute to the development of hypercholesteremia and hyperinsulinemia in *Batf3*$^{-/-}$ mice. (**A**) Schematics of antibiotic treatment of WT and *Batf3*$^{-/-}$ mice. **(B, C)** Fasting serum total cholesterol concentration **(B)**, and serum insulin concentrations **(C)** (NC: n = 4/group; Abx: n = 4-5/group). (**D**) Gating strategy for flow cytometry analysis of IgA coated bacteria. Bacteria were first gated on forward vs. sideward scatter, single cells were gated on FSC-A vs. FSC-H, and bacteria were gated using Sytox green. Data are represented as means ± SD. Statistical significance was determined by Student's *t*-test. *, $p < 0.05$, **, $p < 0.01$, ***, $p < 0.005$.

Figure S7

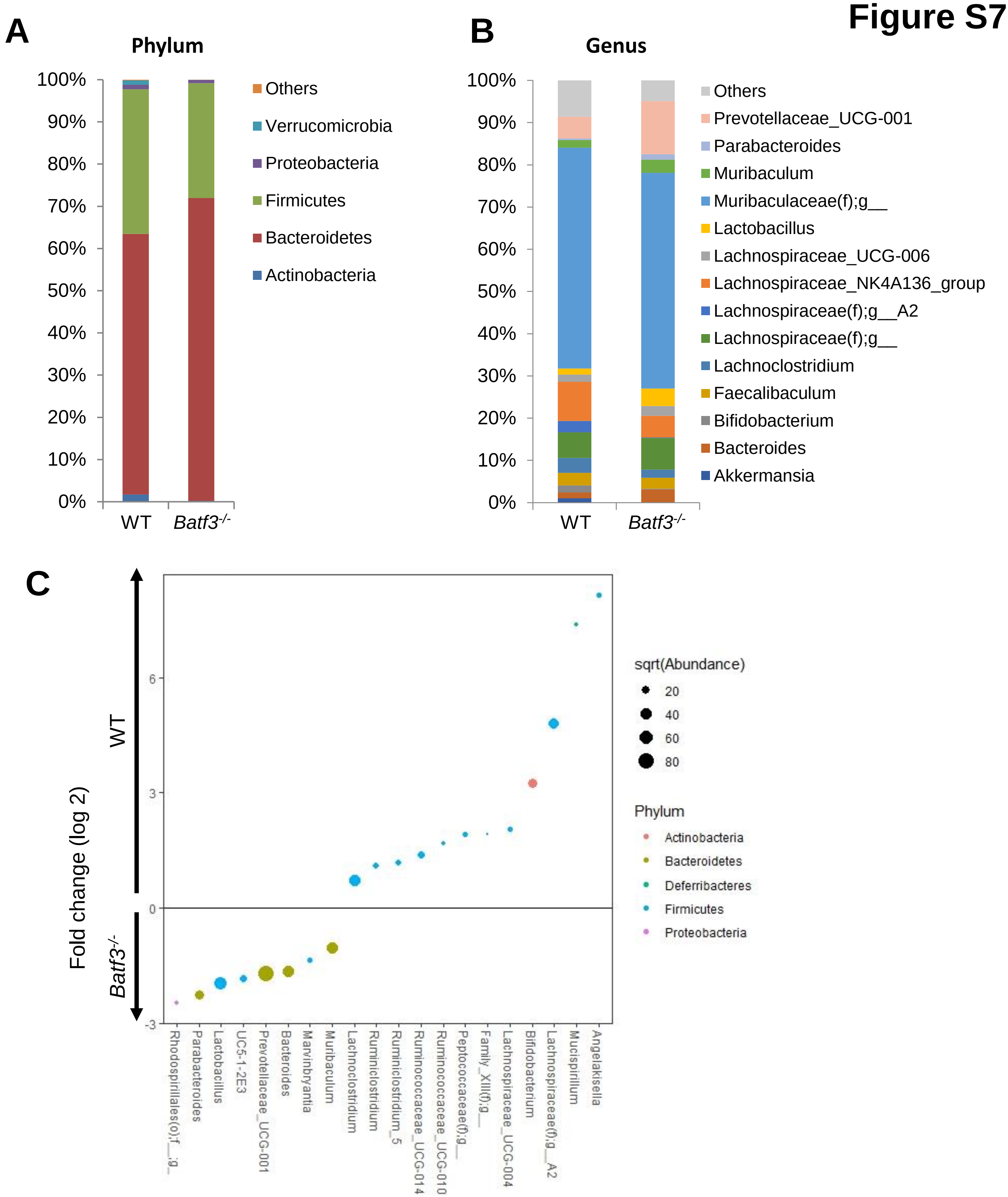
A
Phylum
100%
90%
80%
70%
60%
50%
40%
30%
20%
10%
0%
WT
Batf3-/-
Others
Verrucomicrobia
Proteobacteria
Firmicutes
Bacteroidetes
Actinobacteria
B
Genus
Others
Prevotellaceae_UCG-001
Parabacteroides
Muribaculum
Muribaculaceae(f);g__
Lactobacillus
Lachnospiraceae_UCG-006
Lachnospiraceae_NK4A136_group
Lachnospiraceae(f);g__A2
Lachnospiraceae(f);g__
Lachnoclostridium
Faecalibaculum
Bifidobacterium
Bacteroides
Akkermansia
C
Fold change (log 2)
WT
Batf3-/-
6
3
0
-3
sqrt(Abundance)
20
40
60
80
Phylum
Actinobacteria
Bacteroidetes
Deferribacteres
Firmicutes
Proteobacteria
Rhodospirillales(o);f__g__
Parabacteroides
Lactobacillus
UC5-1-2E3
Prevotellaceae_UCG-001
Bacteroides
Marvinbryantia
Muribaculum
Lachnoclostridium
Ruminiclostridium
Ruminiclostridium_5
Ruminococcaceae_UCG-014
Ruminococcaceae_UCG-010
Peptococcaceae(f);g__
Family_XIII(f);g__
Lachnospiraceae_UCG-004
Bifidobacterium
Lachnospiraceae(f);g__A2
Mucispirillum
Angelakisella

Figure S7: *Batf3*$^{-/-}$ mice harbor a distinct fecal microbial composition compared to WT mice. (A-C) 16s rRNA sequencing was performed on fecal samples from 8-week-old WT and *Batf3*$^{-/-}$ mice (n = 10/ group). (**A**) Relative abundance of bacterial phyla based on number of 16S rRNA reads (%). (**B**) Relative abundances of most abundant genera based on number of reads (%). **(C)** Differential microbial genera were identified by negative binomial models. Log2 of the fold change between *Batf3*$^{-/-}$ and WT is shown. Dot size represents microbial relative abundance and color denotes different phyla. Representation of average bacterial phyla in fecal microbiota of 8-week-old WT and *Batf3*$^{-/-}$ mice.
